# CNS endothelial derived extracellular vesicles are biomarkers of active disease in multiple sclerosis

**DOI:** 10.1186/s12987-021-00299-4

**Published:** 2022-02-08

**Authors:** Michael Mazzucco, William Mannheim, Samantha V. Shetty, Jennifer R. Linden

**Affiliations:** 1 The Brain and Mind Research Institute and the Department of Neurology, Weill Cornell Medical College, 1300 York Ave, New York, NY 10065 USA; 2 Department of Neurology, Weill Cornell Medical College, New York, NY USA

**Keywords:** Multiple sclerosis, Extracellular vesicles, Blood–brain barrier, Endothelial cells, Biomarker

## Abstract

**Background:**

Multiple sclerosis (MS) is a complex, heterogenous disease characterized by inflammation, demyelination, and blood–brain barrier (BBB) permeability. Currently, active disease is determined by physician confirmed relapse or detection of contrast enhancing lesions via MRI indicative of BBB permeability. However, clinical confirmation of active disease can be cumbersome. As such, disease monitoring in MS could benefit from identification of an easily accessible biomarker of active disease. We believe extracellular vesicles (EV) isolated from plasma are excellent candidates to fulfill this need. Because of the critical role BBB permeability plays in MS pathogenesis and identification of active disease, we sought to identify EV originating from central nervous system (CNS) endothelial as biomarkers of active MS. Because endothelial cells secrete more EV when stimulated or injured, we hypothesized that circulating concentrations of CNS endothelial derived EV will be increased in MS patients with active disease.

**Methods:**

To test this, we developed a novel method to identify EV originating from CNS endothelial cells isolated from patient plasma using flow cytometry. Endothelial derived EV were identified by the absence of lymphocyte or platelet markers CD3 and CD41, respectively, and positive expression of pan-endothelial markers CD31, CD105, or CD144. To determine if endothelial derived EV originated from CNS endothelial cells, EV expressing CD31, CD105, or CD144 were evaluated for expression of the myelin and lymphocyte protein MAL, a protein specifically expressed by CNS endothelial cells compared to endothelial cells of peripheral organs.

**Results:**

Quality control experiments indicate that EV detected using our flow cytometry method are 0.2 to 1 micron in size. Flow cytometry analysis of EV isolated from 20 healthy controls, 16 relapsing–remitting MS (RRMS) patients with active disease not receiving disease modifying therapy, 14 RRMS patients with stable disease not receiving disease modifying therapy, 17 relapsing-RRMS patients with stable disease receiving natalizumab, and 14 RRMS patients with stable disease receiving ocrelizumab revealed a significant increase in the plasma concentration of CNS endothelial derived EV in patients with active disease compared to all other groups (*p* = 0.001). Conclusions: For the first time, we have identified a method to identify CNS endothelial derived EV in circulation from human blood samples. Results from our pilot study indicate that increased levels of CNS endothelial derived EV may be a biomarker of BBB permeability and active disease in MS.

**Supplementary Information:**

The online version contains supplementary material available at 10.1186/s12987-021-00299-4.

## Background

Extracellular vesicles (EV) are a heterogeneous group of nanosized vesicles released from all cell types. EV come in a wide range of sizes and compositions but generally refer to three main types of vesicles including exosomes, microvesicles, and apoptotic bodies [1–4]. Generally, exosomes are believed to be ~ 30–150 nm in size, microvesicles ~ 100–1000 nm in size, and apoptotic bodies ~ 1–5 um in size. However, the size and biochemical properties of these vesicles often overlap and the International Society of Extracellular Vesicles (ISEV) strongly discourages classification of EV as exosomes, microvesicles, or apoptotic bodies based on size or protein content [5]. Instead, EV should only be classified as exosomes, microvesicles, or apoptotic bodies only if their mode of biogenesis can be clearly identified. This includes exocytosis of multivesicular bodies for exosome secretion, budding from the plasma membrane for microvesicle secretion, and blebbing from the plasma membrane of apoptotic cells for apoptotic body secretion. Despite their mode of biogenesis, all EV contain a lipid bilayer and a wide array of biologically active components including proteins, lipids, and various nucleic acid species. EV secretion increases when cells are activated or injured and represent the biological state of their parental cell. In addition, EV influence the biological activity of recipient cells and are key regulators of intercellular communication [6–9]. As a result, EV have been implicated as important mediators of both homeostasis and pathogenesis. Because of these properties, EV have gained recent interest as possible biomarkers for numerous diseases including neurological disorders like multiple sclerosis (MS) [10–13].

MS is a chronic, inflammatory disease of the central nervous system (CNS) characterized by demyelination, blood–brain barrier (BBB) permeability, and immune infiltration [14–21]. MS is a complex and heterogeneous disease with various clinical and histopathological classifications [14, 22, 23]. Clinically, MS is classified as relapsing remitting multiple sclerosis (RRMS), secondary progressive multiple sclerosis (SPMS), or primary progressive multiple sclerosis (PPMS). RRMS, the most common type, is defined by acute episodes of active disease (relapses) followed by periods of complete or partial recovery (remission) [24–26]. Within ten to twenty years after disease onset, most RRMS patients transition to SPMS characterized by worsening neurologic decline without recovery. In comparison, PPMS is less common and is characterized by progressive neurological decline without recovery from disease onset. Active disease is characterized by acute neurological decline and/or detection of new lesion formation via MRI and can occur in all clinical states but is most common in RRMS.

Currently, MS disease activity is typically monitored by detection of contrast enhancing lesions (CEL) via MRI [25, 27]. On histopathological examination, CEL exhibit overt BBB permeability, active demyelination, and pronounced inflammation [28–30]. Enhancement usually resolves within two to eight weeks, highlighting the transient nature of these lesions. However, due to costs, logistics, and possible safety concerns over repetitive use of contrast reagents, MRI are typically only performed annually after disease diagnosis. Consequently, detection of ongoing disease activity is often missed, emphasized by the detection of new, but non-enhancing lesions on annual patient MRI. Delayed detection of active disease may have negative consequences for patients including delayed treatment and accumulation of lesions that may influence worse clinical outcomes [31–33]. As a result, disease monitoring would greatly benefit from identification of an easily accessible biomarker of disease activity. We believe that EV, especially CNS derived EV, are excellent candidates to fulfill this need.

The use of EV as MS biomarkers has gained recent interest [10–13]. Most of the studies focusing on EV as possible biomarkers have concentrated on EV isolated from peripheral blood (serum or plasma) or cerebral spinal fluid (CSF). Of these studies, EV isolated from CSF have demonstrated good correlation with disease activity [34–39]. However, collection of CSF requires an invasive lumbar puncture with rare, but serious complications and significant patient anxiety [40–43]. In comparison, EV isolated from peripheral blood use minimally invasive techniques that can be performed regularly. Although, previous studies examining EV isolated from peripheral blood have revealed differences between healthy controls and MS patients, these studies are limited as most do not focus on CNS derived EV and therefore may not be specific markers of neurological pathogenesis [44–55]. Analysis of CNS derived EV, in comparison, may identify specific neurological maladies and elucidate the molecular mechanisms involved in disease pathogenies. Under normal circumstance, the human CNS is inaccessible to experimental investigation. However, because EV represent the biological state of the cell they originate from, molecular examination of CNS derived EV isolated from peripheral blood may allow a unique opportunity to study the molecular state of CNS cells in real-time, in living patients.

In this study, we propose to evaluate the circulating concentrations of EV derived from CNS endothelial cells as a biomarker of active disease and BBB dysfunction in MS patients. CNS endothelial cells are a key component of the BBB and play a central role in MS pathogenesis [56–60]. Because BBB permeability is a key step in MS pathogenesis, assessment of CNS endothelial cell activation or injury via EV analysis may help elucidate key processes in MS lesion development. To identify EV specifically originating from CNS endothelial cells, we will examine EV isolated from patient plasma for expression of pan-endothelial markers CD31 (PECAM-1), CD105 (Endoglin), or CD144 (VE-Cadherin) [61]. To determine if EV positive for CD31, CD105, or CD144 originate from CNS endothelial cells, we will examine EV for expression of the myelin and lymphocyte protein MAL, which has been demonstrated to be specifically expressed by CNS endothelial cells versus endothelial cells of peripheral organs in mice [62, 63] and whose expression in human brain endothelial cells has been confirmed by transcriptional analysis [64–66]. We will compare circulating EV levels between healthy controls (HC), RRMS patients with active disease not receiving disease modifying therapy (DMT), RRMS patients with stable disease not receiving DMT, RRMS patients with stable disease receiving natalizumab, and RRMS patients with stable disease receiving ocrelizumab. We hypothesize that circulating concentrations of CNS endothelial derived EV (CNS-EEV) will be higher in RRMS patients with active disease compared to healthy controls or stable RRMS patients.

## Methods

### Subject recruitment

Study subjects were recruited and consented according to Weill Cornell Medicine Institutional Review Board–approved protocol 1003010940. Subjects were recruited at The Judith Jaffe Multiple Sclerosis Clinical Care and Research Center between 2017–2019. Study subjects included individuals with clinically definite MS according to the 2010 McDonald criteria [25, 67] and HC without MS. Active RRMS patients were identified by recent physician-confirmed clinical relapse and/or detection of a CEL via MRI within two months of specimen collection. RRMS patients were considered DMT negative if they were DMT naïve or had not received DMT for at least one year prior to specimen collection. For inclusion into the natalizumab or ocrelizumab groups, patients were required to be on DMT for at least one week prior to specimen collection. Disease duration was determined from estimated date of first symptoms to specimen collection. Expanded Disability Status Scale (EDSS) scores were calculated by physicians within three months of specimen collection. Patient demographics are summarized in Table 1.

**Table 1:** Demographics and clinical characteristics of study participants

	HC	Active	Stable	NTZ	OCZ
	*n* = 20	*n* = 16	*n* = 14	*n* = 17	*n* = 14
Age, mean, SD, y	37(13)^*a*^	36(10)	43(12)	39(10)	41(11)
% Female (n)	75% (15)*	69% (11)	93%(13)*	94%(16)*	64%(9)
Clinical State		RRMS	RRMS	RRMS	RRMS
Disease State		Active^*b*^	Stable	Stable	Stable
DD, median (IQR), mo		22.5(2.5–56)^#^	142.5(62.5–183)	87(47.5–157.5)	159.5(33–239.3)^#^
EDSS, median, (IQR)		1.5(0.3–2.4)	1.5(0.0–4.1)	1(0.0–3.0)	3.3(1.5–4.5)

*DD* disease duration, *EDSS* Expanded Disability Status Scale, *IQR* interquartile range, *m* month, *SD* standard deviation, *y* year

^*a*^One undetermined value, not included in correlation analysis

^*b*^14 out of 16 (87.5%) patients have confirmed contrast enhancing lesion

^*^p < 0.002 HC vs. stable, HC vs. NTX, determined by Fishers exact test between pairs

^#^*p* = 0.007 Active vs. OCZ, determined by ANOVA with Tukey's multiple comparisons test

### Blood collection and plasma separation

Detailed methodology can be found in the Additional file 1. Briefly, peripheral blood was harvested via venipuncture into K2 EDTA vacutainers and stored at room temperature (RT) up to six hours (h) until processing. Platelet free plasma (PFP) was prepared by centrifuging vacutainer tubes at 450 g for ten minutes at RT. Plasma was transferred to 15 mL conical tubes and centrifuged twice at 2000 g for ten minutes (m) at RT to pellet platelets. PFP was separated into 1 mL aliquots and stored in 2.0 mL cryogenic vials at -80 °C. Prior to use, 1 mL aliquots were thawed in 37 °C water bath for two minutes (m).

### EV experimentation

Detailed methodology for EV experimentation can be found in the Additional file 1. Below are brief explanations of the methodology used. Only 0.2 μm filtered phosphate buffered saline (PBS) and flow cytometry sheath fluid (BD FACSflow) were used for experiments.

### EV isolation from PFP via size exclusion chromatography (SEC)

Izon qEV 70 nm size exclusion columns (SEC) were equilibrated to RT and washed with 10 mL of PBS. These columns have an optimum recovery of particles ranging in size from 70 to 1000 nm. To collect SEC fractions, 1 mL of PFP was applied, allowed to fully absorb, and then eluted with 6 mLs of PBS. Fractions were collected in twelve, 0.5 mL, sequential aliquots. For patient analysis, fractions seven, eight, and nine were pooled together for a total of 1.5 mL SEC isolated EV in PBS. Selection of these fractions is consistent with previously published results using qEV SEC to isolate EV from plasma [68–73].

### Transmission electron microscopy (TEM) analysis

SEC isolated EV in PBS were applied to Glow-discharge Formvar and carbon-coated 400 mesh copper grids (Electron Microscopy Sciences) and stained with 1.5% aqueous uranyl acetate. Samples were viewed on a JEM-1400 transmission electron microscope (JEOL) operated at 100 kV and images were captured on a Veleta 2 K x 2 K CCD camera (EM-SIS).

### Nanoparticle tracking analysis (NTA)

SEC isolated EV in PBS were characterized by NTA using a Malvren NanoSight NS500 instrument equipped with an Andor EM-CCD camera. Video acquisitions were performed with NTA software v3.1.

### Western blot analysis

SEC isolated EV were centrifuged at 100,000 g for one h at 4 °C. The EV pellet was lysed in radioimmunoprecipitation assay buffer (Thermo Scientific) containing Halt Protease and Phosphatase Inhibitor (Thermo Scientific) for ten minutes on ice and stored a − 20 °C until use. Protein concentration was determined by Pierce BCA Protein Assay Kit (Thermo Scientific). Human umbilical cord vein endothelial cell (HUVEC) lysate (Novus Biologicals) was used as a control. 2 μg of lysates were diluted into 4 × Laemmli Sample Buffer (Bio-Rad) containing 5% 2-Mercaptoethanol and heated at 95 °C for five m and loaded onto 4–20% Mini-PROTEAN TGX Stain-Free gels (Bio-Rad) then transferred to nitrocellulose membranes using the Bio-Rad Trans-Blot SD Semi-Dry Electrophoretic Transfer Cell system (Bio-Rad). Membranes were blocked with 5% Blotting-Grade Blocker nonfat milk (Bio-Rad) in Tris Buffered Saline with Tween 20 (TBS-T) (Bio-Rad) blocking solution for one h at RT and then incubated with anti-flotillin, CD9, CD63, CD31, CD105, and CD144 antibodies in blocking solution overnight at 4 °C as indicated in Additional file 1: Table S2. Blots were washed with TBS-T and then incubated with secondary antibody peroxidase-conjugated Affinipure Goat Anti-Rabbit IgG H + L (Jackson ImmunoResearch) at 0.024 µg/mL in blocking solution for two h at RT, washed in TBS-T, and developed for five m at RT in Clarity Max Western ECL Substrate (Bio-Rad). The developed blots were visualized on 5 × 7 CL-XPosure Films (Thermo Fisher Scientific) at various exposure times using a Konica Minolta SRX-101A film processor.

### Centrifugation experiments

250 μL aliquots of SEC isolated EV in PBS were centrifuged at 18,000 g or 100,000 g for one h at 4 °C. Supernatants were carefully removed and analyzed via flow cytometry. EV pellets were vigorously resuspended in 250 μL PBS and analyzed via flow cytometry and TEM.

### Preparation of fluorescently labeled MAL ligand

Epsilon protoxin (pETX), from *Clostridium perfringens*, Strain 34 Type B, was provided by Biodefence and Emerging Diseases Resources at a minimum > 95% purity at 0.5 mg/ml. pETX was labeled with Life Technologies Alexa Fluor 647 Protein Labeling Kit per the manufacturer's instructions (pETX-647). Labeled toxin (~ 11 μM) was stored at 4 °C until use.

### Flow cytometry settings for analysis of submicron particles

**Fig. 1:**
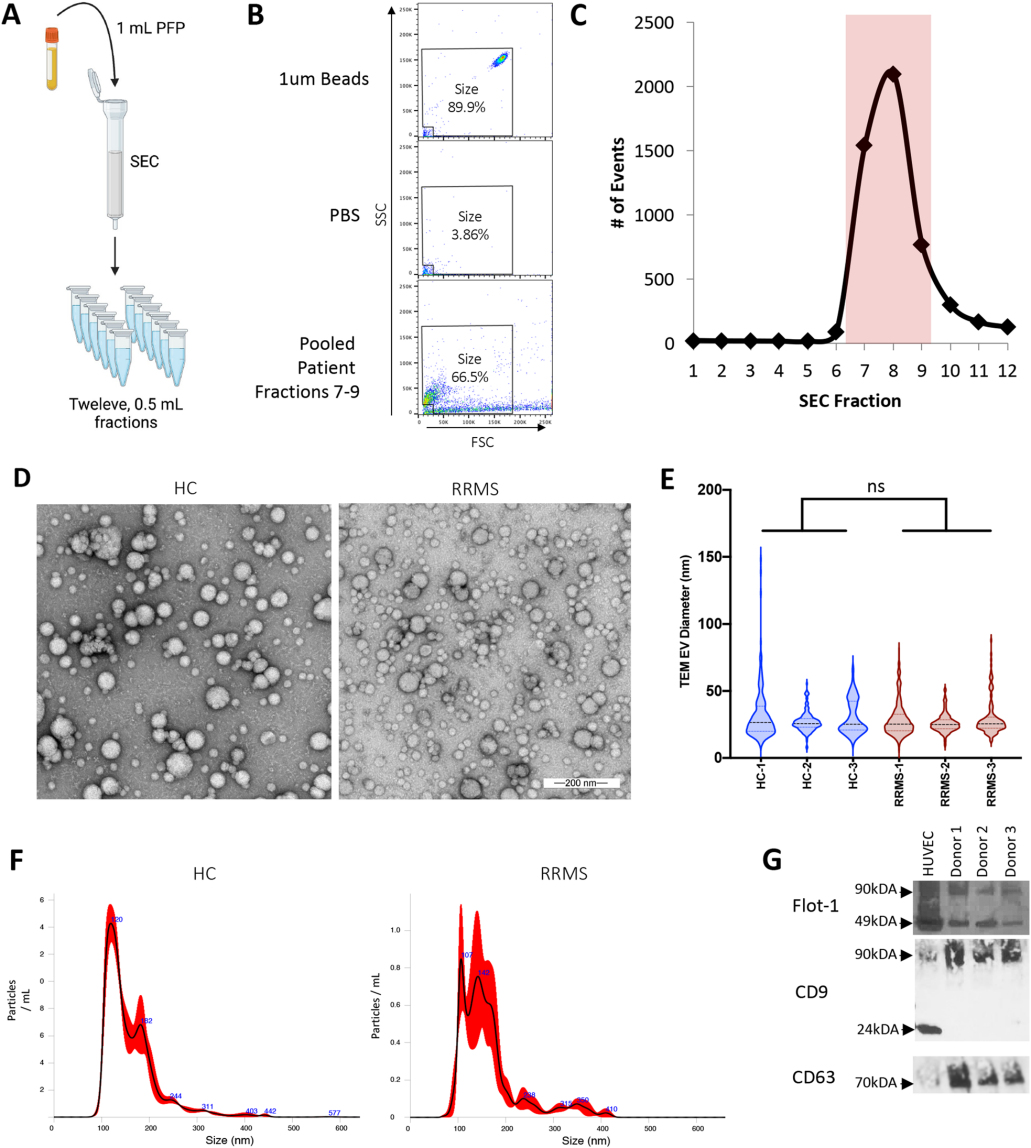
Identification of SEC fractions for EV analysis via flow cytometry. **A** Schematic of experimental design. Twelve, 0.5 mL aliquots were collected after 1 mL of PFP was applied to SEC. **B** SEC fractions were analyzed via flow cytometry after FSC and SSC voltage was adjusted to reliably detect 1 μm beads (polystyrene microspheres) (top) and excluding mechanical noise observed in PBS alone (middle). Example of pooled SEC fractions seven, eight, and nine pooled from an individual donor (bottom). **C** The number of events within the size gate for each individual 0.5 ml SEC fractions was determine via flow cytometry. Fractions seven, eight, and nine (highlighted in red) contained the majority of detectable events and were pooled together for future analysis. **D** TEM analysis of pooled fractions from HC and RRMS patient. **E** Violin plots of EV diameter measured from TEM images for three HC and three RRMS patients. ns = nonsignificant determined by t-test. **F** Representative examples of NTA results of EV isolated from a HC and RRMS patient. **G** Western blot analysis of indicated proteins from purified EV lysates from three separate donors. HUVEC lysate was used as a positive control

**Fig. 2:**
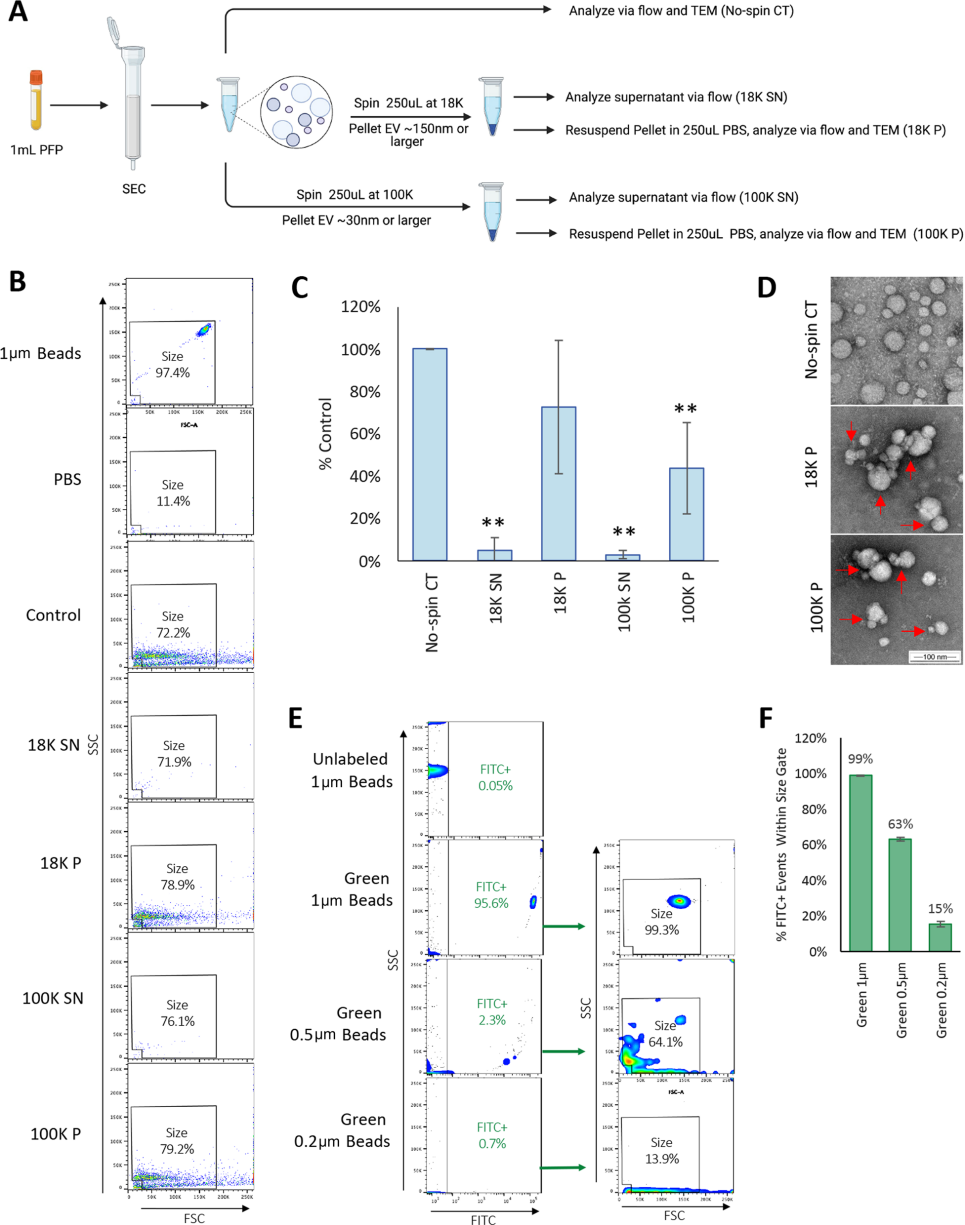
Determination of flow cytometry size resolution limits. **A** Schematic of experimental approach for high-speed centrifugation experiments. EV from 1 mL PFP were isolated in 1.5 mL PBS using SEC as described. A control aliquot (no spin CT) was immediately analyzed via flow cytometry and TEM. Equal portions of isolated EV in PBS (250 uL) were centrifuged at 18,000 g (18 K) or 100,000 g (100 K) for one h to pellet large EV (~ 150 and larger) or small EV (~ 30 nm and larger), respectively. Supernatants (SN) were harvested, and EV enumerated via flow. EV Pellets (P) were resuspended in 250 uL PBS and then analyzed via flow and TEM. **B** Representative dot plots for each condition using size gate. 1um beads (polystyrene microspheres) and PBS are shown as controls. **C** Counts were normalized to no-spin controls and expressed as percent control. Results represent the mean ± STDEV of four separate donors. ** *p* < 0.01 versus control determined by one-way ANOVA with post-hoc Tukey HSD Test. **D** TEM analysis of EV in no-spin controls or after resuspension from 18 and 100 K pellets. Red arrows point to EV aggregates observed in resuspended EV pellets. **E** Flow cytometry analysis of unlabeled 1, 0.5, and 0.2 μm green-fluorescent beads (polystyrene microspheres). Unlabeled 1 μm beads were used as negative controls. Events positive for green fluorescence measured on the FITC channel (FITC +) were analyzed using the size gate determined in previous experiments. **F** Quantification of the percentage of FITC + events detected within the size gate. Results represents the mean ± STDEV of three replicates

All samples were analyzed using a BD FACSVerse flow cytometer with a three-laser configuration (488, 640, and 405 nm) equipped with a BD Flow sensor using the manufacturer settings. BD FACSuite CS&T research beads were used daily to calibrate and perform quality control checks per the manufacturer’s instructions. All samples were analyzed in 5 mL polystyrene round bottom tubes using a medium sample flow rate (60 μL / m) with a normal sheath core stream fluid velocity (5.5 m / s). Data was collected using BD FACSuite software and exported to BD Flowjo software for analysis. Event counts and volume metrics were exported to Microsoft Excel for final calculations. For submicron analysis, forward scatter (FSC) and side scatter (SSC) voltage was adjusted to 765 and 465, respectively, with a FSC threshold of 10,000. Settings were evaluated using Invitrogen’s Flow Cytometry Size Kit Calibration Kit containing nonfluorescent polystyrene microspheres ranging in size from 1 to 15 μm and Invitrogen’s Flow Cytometry Submicron Particle Size Reference Kit containing green-fluorescent polystyrene microspheres ranging in size from 0.02 to 2 μm. Quality control checks for the size gate was selected using Invitrogen’s 1 μm nonfluorescent polystyrene microspheres (Fig. 1). Quality control experiment indicate that our size gate identifies events ranging in size from 0.2 to 1 μm (Fig. 2). To determine the appropriate dilution to minimize swarm detection using our flow cytometer, we performed serial dilutions with both polystyrene beads and isolated EV [68, 74, 75]. These results indicated that a concentration of 50 events / μL resulted in reliable detection of single events within our size gate (data not shown). This final concentration of 50 events / μL were used for all subsequent EV analysis. Finally, when EV samples were treated with various detergents to induce EV lysis, there was a marked reduction in detectable events, indicating that the events we are detecting are consistent with EV (data not shown) [73, 76, 77].

### Staining protocol for flow cytometry analysis

The concentration of SEC isolated EV in PBS was determined via flow cytometry using the size gate determined with 1 μm beads as previously described. Isolated EV were adjusted to 500 events / μL and stained with pETX-647, anti-CD3, CD9, CD31, CD41, CD63, CD81, CD105, and CD144 antibodies, and isotype controls as indicated in Additional file 1: Table S1 for two h at RT. As a negative control, pETX-647 was pretreated with an anti-ETX neutralizing antibody, JL004 [78], at 1 mg/mL for 20 m at 37 °C to prevent binding. Prior to flow cytometry analysis, stained EV were diluted with 500 μL of PBS for a final concentration of 50 events / μ to prevent swarming artifacts during analysis. To determine gates positive for expression, EV stained with isotype controls were used as negative controls for each individual donor (Additional file 1: Fig. S1A–D). To determine gates positive for MAL expression, EV stained with pETX-647 pretreated with JL004 (Fig. 4A) were used as negative controls for each individual donor.

### Statistics

When comparing two data points, unpaired student’s t-tests were used to determine significance. When comparing three or more data points, one-way ANOVA with post-hoc Tukey HSD test was used to determine significance. These instances are indicated in figure legends. Pearson’s correlation analyses as well as paired t-tests were performed where indicated in figure legends. D'Agostino's K-squared test was used to test for normality in the different patient groups using the total CNS-EEV concentrations as described in Fig. 6D. All patient groups including HC, active RRMS patients not receiving DMT (Active), stable RRMS patients not receiving DMT (Stable), and stable RRMS patients receiving natalizumab (NTZ) passed the normality test (alpha = 0.05). However, RRMS patients receiving ocrelizumab (OCZ) did not pass (*p* = 0.006).

## Results

### Study cohort

We enrolled a total of 20 HC, 16 RRMS patients with active disease not receiving DMT, 14 RRMS patients with stable disease not receiving DMT, 17 RRMS patients with stable disease receiving natalizumab, and 14 RRMS patients with stable disease receiving ocrelizumab (Table 1). Active disease was determined by recent physician-confirmed relapse and/or detection of a CEL via MRI. Of the 16 RRMS patients with active disease, 14 had confirmed CEL (87.5%). Donors with significant comorbidities known to alter circulating EV concentrations including cancer, other autoimmune diseases, morbid obesity (body mass index of 40 or more), diabetes mellitus type 2, or recent cardiovascular or cerebrovascular events were excluded [79–84]. Subject groups were comparable in age however the sex ratio varied. EDSS scores were comparable between RRMS groups however disease duration (DD) was varied.

### Characterization of SEC isolated EV

To determine the optimal collection procedure for isolation of EV via SEC for flow cytometry analysis, twelve, 0.5 mL fractions were collected via SEC after addition of 1 mL PFP (Fig. 1A). To detect submicron events via flow cytometry, we used 1 μm beads (polystyrene microspheres) diluted in PBS to select appropriate flow cytometry settings (Fig. 1B). A size gate was designated based on 1 μm beads (polystyrene microspheres) and excluded mechanical noise observed in PBS alone. Analysis revealed that SEC fractions seven, eight, and nine contained the highest number of events within our designated size gate (Fig. 1C). This is consistent with previously published results using the same SEC to isolate EV from plasma samples[68–73]. Based on these results, fractions seven, eight, and nine were pooled together for all subsequent analysis.

### Determination of flow cytometry size resolution

Because conventional flow cytometry can typically only detect submicron particles 0.2 – 0.7 μm or larger [85–87], we wished to further characterize our SEC isolated samples using TEM analysis, NTA analysis, and western blot analysis (Fig. 1D–G). Samples isolated from HC and RRMS were compared for thoroughness. TEM analysis of the pooled aliquots from HC and RRMS patients revealed spherical particles with morphologies indicative of EV (Fig. 1D). Although there was a wide range of particle diameters observed via TEM, most particles observed were approximately ~ 30 nm in diameter. In addition, there was no significant difference in the mean size of particles between HC or RRMS patients, 30 and 28 nm, respectively (Fig. 1E). However, it should be noted that TEM may underestimate EV size due to dehydration during fixation [69, 85, 86]. In comparison, NTA analysis, which reliable detect particles ranging in size from ~ 80 nm to 0.4 μm [5, 85, 86], revealed a mean particle size of 160 nm and 165 nm for HC and RRMS patients, respectively (Fig. 1F). Finally, western blot analysis of pelleted EV revealed the presence of EV related proteins including flotillin-1, CD9, and CD63 (Fig. 1G). Taken together, this data indicates that our SEC isolated EV contain a heterogenous population of EV, consistent with previous results [68–73, 88–90].

Although it is accepted that conventional flow cytometry can detect submicron particles 0.2–0.7 μm or larger [85–87], we wished to better define the size resolution of our flow cytometry size gate. To achieve this, we first completed a series of experiments using high speed centrifugation (Fig. 2A–D). It is well accepted that centrifugation between 10 and 20,000 g causes sedimentation of larger EV (~ 150 nm and larger) while centrifugation at 100,000 g causes sedimentation of smaller EV (~ 30–150 nm and larger)[91–93]. To determine if our flow cytometry settings detected small or large EV, isolated EV were centrifuged at 18,000 g for one h to pellet large EV and 100,000 g for one h to pellet small EV (Fig. 2B and C). Supernatants were harvested and analyzed via flow cytometry and concentrations compared to samples that were not centrifuged (no-spin controls). EV pellets were resuspended in the same volume of PBS as the starting material prior to analysis. Results revealed that the 18,000 g and 100,000 g supernatants contained 5% and 3% of events compared to no-spin controls, respectively (Fig. 2C), indicating that flow cytometry analysis is detecting EV larger than ~ 150 nm. These results were confirmed when examining the number of events detected in the resuspended pellets. The 18,000 g and 100,000 g resuspended pellets contained 73% or 44% of events compared to no-spin controls, respectively. The reduction in events seen in the resuspended pellets is most likely due to EV aggregation, as observed via TEM (Fig. 2D). This aggregation was not observed in the no-spin controls. Taken together, this data indicates that our size gate is detecting EV larger than 150 nm.

To further define our size resolution via flow cytometry, we performed an experiment using an array of differently sized fluorescent-green submicron beads (polystyrene microspheres) (Fig. 2E and F). 1, 0.5 and 0.2 μm green-fluorescent beads were analyzed via flow cytometry using the settings described above. Unlabeled 1 μm beads were used as a negative control. By selecting for events with positive green fluorescence compared to unlabeled 1μm beads, we determined that our size gate detects 99% of 1 μm green-fluorescent beads, 63% of 0.5 μm green-fluorescent beads, and 15% of 0.2 μm green-fluorescent beads (Fig. 2F). This data indicates that our size gate detects events ranging in size from 0.2 to 1 μm, with the majority of events ranging in size from 0.5 to 1 μm. This is consistent with previous results indicating conventional flow cytometers can detect submicron particles ranging in size from 0.2 to 0.6 μm or larger [85–87]. However, it should be noted that polystyrene beads have a higher refractive index compared to EV, making direct, comparative size estimations between the two nanoparticles difficult [94–96].

### Characterization of EV isolated from patient plasma

**Fig. 3:**
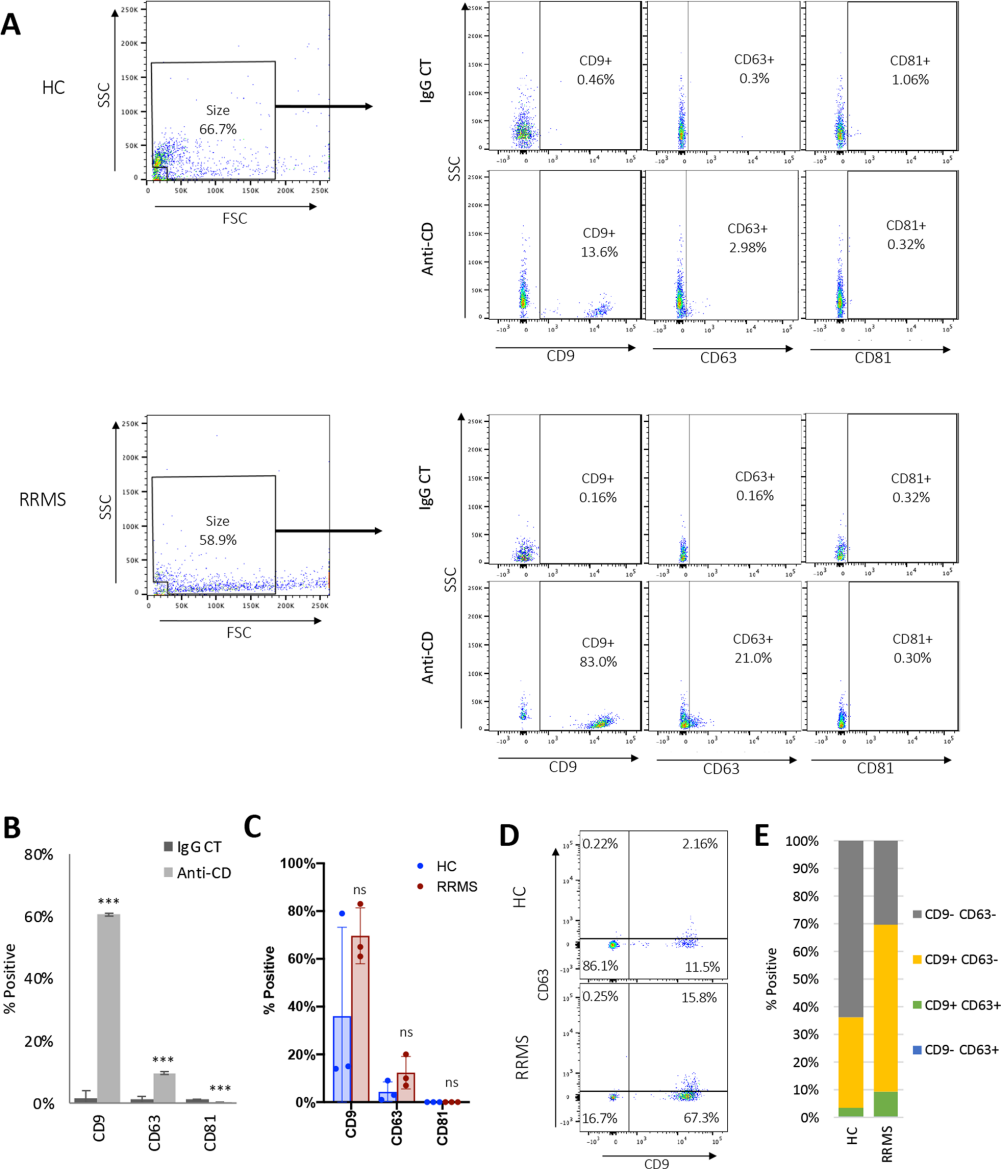
Characterization of EV isolated from patient plasma samples. **A** SEC isolated EV were examined for expression of common EV markers CD9, CD63, and CD81 via flow cytometry. Representative dot plots from a healthy control (HC) and RRMS patient. FSC and SSC was used to select events of appropriate size using the size gate determined as previously described. Events from the size gate were analyzed for expression for expression CD9, CD63, or CD81 via staining with corresponding anti-CD antibodies (anti-CD). EV stained with appropriate isotype control (IgG CT) were used as negative controls. Positive gates were determined using IgG-stained controls for each individual donor. **B** Representative results from a single donor for the percentage of size events positive for indicated surface markers when stained with anti-CD markers or isotype controls (IgG CT). Results represents the mean ± STDEV of four replicates. *** *p* ≤ 0.001 determined by t-test. **C** Percentage of size events positive for indicated surface markers from HC or RRMS patients. Results represents the mean ± STDEV of three separate donors. Each dot is an individual donor. ns = not significant determined by t-test. **D** Representative dot plots comparing size events positive for CD9 or CD63 isolated from a HC or RRMS patient. **E** Percentage of size events negative for CD9 and CD63 (CD9−, CD63−), positive for CD9 and negative for CD63 (CD9+ , CD63−), positive for CD9 and CD63 (CD9+ , CD63+) or negative for CD9 and positive for CD63 (CD9−, CD63+) from HC or RRMS patients. Results represents the mean of three separate donors. Percent of events were not significantly different between HC and RRMS patients as determined by t-test

Our results indicate that our flow cytometer was able to detect submicron particles ranging in size from 0.2 to 1 μm using the size gate described above (Fig. 2). To determine if the events within our size gate were consistent with EV, SEC isolated EV were analyzed for EV specific markers CD9, CD63, and CD81 via flow cytometry (Fig. 3A–F). SEC isolated EV were stained with fluorescently conjugated anti-CD antibodies and appropriate isotype controls (Fig. 3A and B). EV isolated from three HC and three RRMS patients were evaluated for comparison purposes only. When staining for CD9, a significantly higher percent of EV stained positive for CD9 versus the isotype control, 60.7 versus 1.5%, respectively. When staining for CD63, a significantly higher percent of EV stained positive for CD63 versus the isotype control, 9.7 versus 1.3%, respectively. In comparison, when staining for CD81, a significantly lower percent of EV stained positive for CD81 versus the isotype control, 0.1 versus 1.23%, respectively. Taken together this data indicates EV within our size gate are positive for CD9 and CD63, but not CD81. Analysis of EV isolated from three HC and three RRMS patients revealed that most events within the size gate expressed CD9 (14.8–78.7%) while a smaller portion expressed CD63 (1.4–9.7%) (Fig. 3C). When comparing the percentage of size events positive for CD9, CD63, or CD81 between HC and RRMS patients, no difference was observed. When examining size events for dual expression of CD9 and CD63, the majority of events either expressed CD9 only (CD9+ CD63-, 14.0–71.7%) or none (CD9- CD63-, 21.2–84.9%) (Fig. 3D and E). A small percentage of events were positive for both CD9 and CD63 (CD9 + CD63 + , 0.8–7.6%) and very few events were positive for CD63 only (CD9- CD63+ , 0.2–0.4%). No significant difference was observed between HC or RRMS patients. As noted previously, comparisons between three HC and three RRMS patients were performed for descriptive purposes only and not powered to reveal significant differences between HC and RRMS patients. Instead, these experiments were designed to demonstrate that the events detected within our size gate are consistent with EV by detection of common EV markers. Taken together, this data indicates that our flow cytometry methods are successfully detecting events consistent with EV as demonstrated by CD9 and CD63 expression.

### EV isolated from patient plasma express the Myelin and Lymphocyte protein MAL

**Fig. 4:**
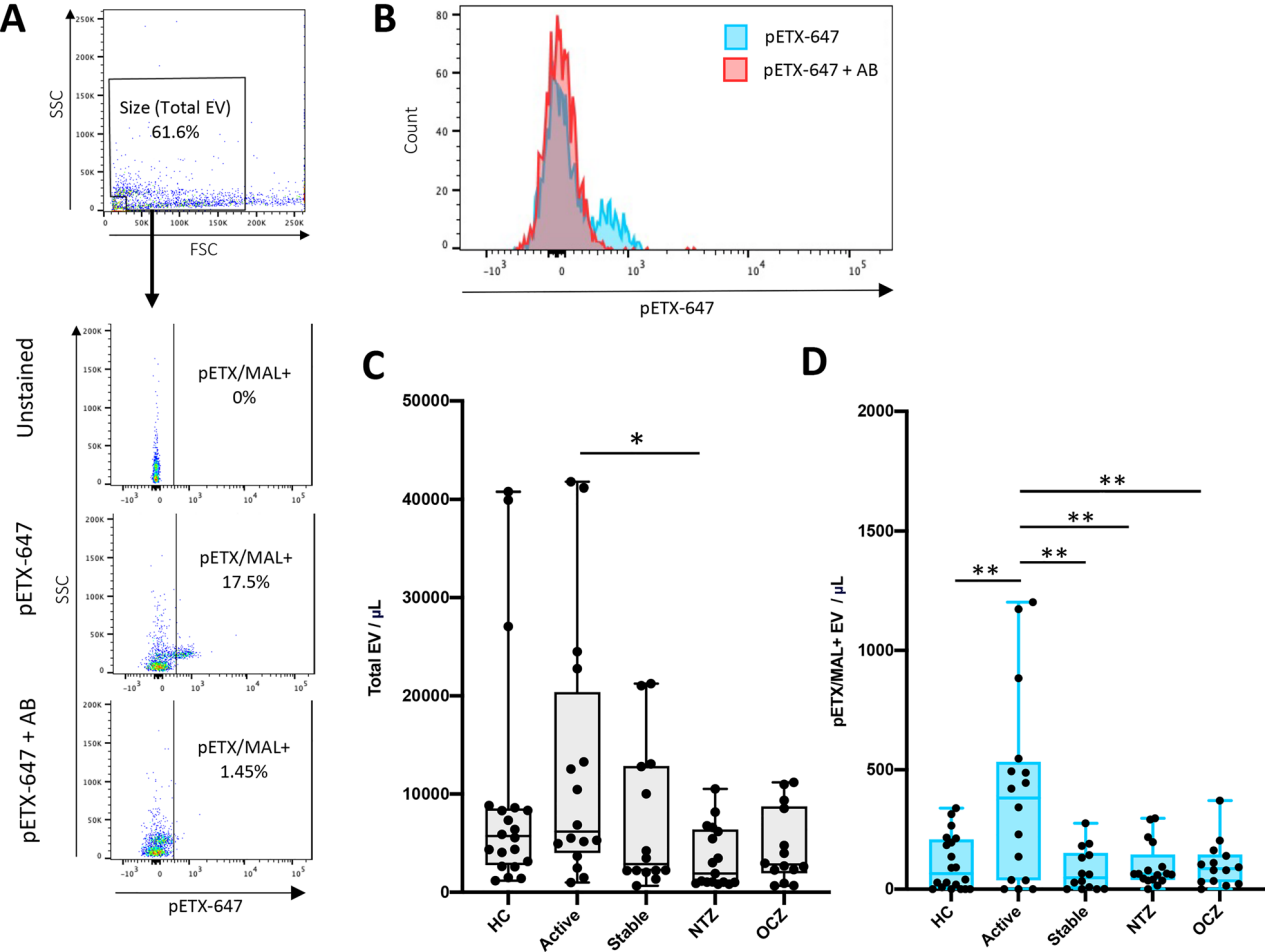
Detection of MAL expression on EV by pETX-647 binding. **A** Gating strategy for identification of pETX/MAL+ EV. SEC isolated EV were probed with pETX-647 or pETX-647 pretreated with a neutralizing antibody (pETX-647 + AB) which inhibits pETX-647 binding to MAL as a negative control. Unstained EV were used as an additional control. EV of appropriate size were analyzed for pETX-647 binding. **B** Histogram representation of pETX-647 fluorescence when EV are stained with pETX-647 or pETX-647 pretreated with the neutralizing antibody (pETX-647 + AB). **C** Enumeration of total EV identified from the size gate per μL of plasma for different patient groups including HC, active RRMS patients not receiving DMT (Active), stable RRMS patients not receiving DMT (Stable), or stable RRMS patients receiving natalizumab (NTZ) or ocrelizumab (OCZ). **D** Enumeration of pETX/MAL+ EV concentrations from indicated patient groups. Results are displayed as box and whisker plots with each individual patient represented as a dot. * *p* ≤ 0.05, ** *p* ≤ 0.01 determined by one-way ANOVA with post-hoc Tukey HSD Test

We have previously demonstrated that CNS endothelial cells specifically express MAL compared to endothelial cells from other organs as determined by binding of the MAL-specific ligand, *Clostridium perfringes* epsilon protoxin (pETX) in mice [62]. To identify EV originating from CNS endothelial cells, we have proposed to detect a combination of pan-endothelial markers (CD31, CD105, or CD144) and MAL on individual EV. However, we first wished to determine if EV MAL expression was detectable via flow cytometry using the MAL-specifc ligand, pETX [97, 98]. To detect MAL expression, SEC isolated EV were probed with Alexaflour-647 conjugated pETX (pETX-647). As a negative control, EV were also probed with pETX-647 pretreated with an antibody that prevents pETX binding to MAL [78] (Fig. 4A). Unstained EV were used as an additional control. Staining with pETX-647 demonstrated a significant increase in fluorescence compared to EV stained with the antibody treated pETX-647 (Fig. 4B). This data indicates that MAL expression on EV can be detected by pETX-647 binding.

The concentration of total EV (Fig. 4C) and the concentration of pETX/MAL + EVs (Fig. 4D) were then determined for our different patient groups. Enumeration of total EV concentrations revealed a significant difference in concentrations between RRMS patients with active disease and RRMS patients receiving natalizumab, but no other significant differences were observed (Fig. 4C). In comparison, when examining concentrations of pETX/MAL+ EV, active RRMS patients had significantly higher concentrations than all other patient groups (Fig. 4D). This data indicates pETX/MAL+ EV concentrations are increased in RRMS patients with active disease. Taken together, this data indicates that our proposed phenotyping stratergy to identify EV originating from CNS endothelial cells may be successful.

### CNS-EEV concentrations are increased in active MS

**Fig. 5:**
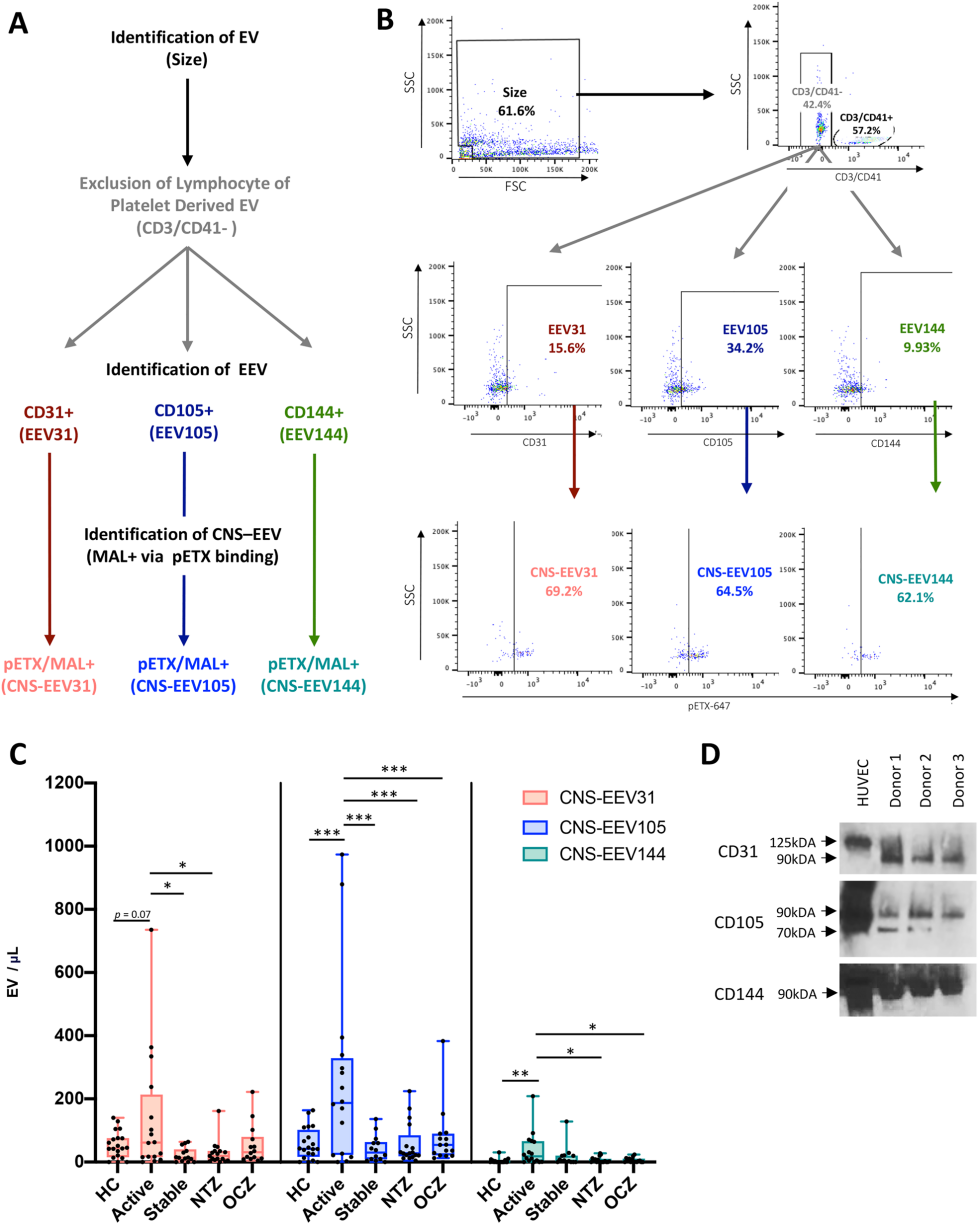
Phenotyping Strategy and enumeration of CNS-EEV from different patient populations. Diagram (**A**) and representative dot plots (**B**) of gating strategy to detect EV derived from CNS endothelial cells. EV of appropriate size were analyzed for the presence of CD3 or CD41 to determine if they were of lymphocyte or platelet origin, respectively. EV negative for CD3 and CD41 (CD3/CD41-) were analyzed for the presence of pan-endothelial markers CD31, CD105, and CD144. EV positive for CD31, CD105, or CD144 are referred to as EEV31, EEV105, and EEV144, respectively (Reviewed in Table 2). To determine if EEV31, EEV105, and EEV144 are derived from CNS endothelial cells, these events were analyzed for the expression of MAL via pETX-647 binding. EEV31, EEV105, and EEV144 positive for pETX/MAL are referred to as CNS-EEV31, CNS-EEV105, and CNS-EEV144, respectively (Reviewed in Table 2). Representative scatter plots of anti-CD-stained EV and their isotype controls are depicted in Additional file 1: Fig. S1. **C** Enumeration of CNS-EEV31, CNS-EEV105, and CNS-EEV144 per μL of plasma from different patient groups including HC, active RRMS patients not receiving DMT (Active), stable RRMS patients not receiving DMT (Stable), or stable RRMS patients receiving natalizumab (NTZ) or ocrelizumab (OCZ). Results are displayed as box and whisker plots with each dot representing an individual patient. * *p* ≤ 0.05, ** *p* ≤ 0.01, *** *p* ≤ 0.001 determined by one-way ANOVA with post-hoc Tukey HSD Test. **D** Western blot analysis of EV lysates for CD31, CD105, and CD144. HUVEC lysate was used as controls

To identify EV originating from CNS endothelial cells (CNS-EEV), we developed a phenotyping strategy to identify EV that express both pan-endothelial markers CD31, CD105, or CD144 and MAL within our designated size gate (Fig. 5A and B). Representative scatter plots of anti-CD-stained EV and corresponding isotype controls are depicted in Additional file 1: Fig. S1A–D. Because platelets also express CD31, we excluded platelet derived EV by eliminating events positive for CD41. Because lymphocytes also express MAL, we also excluded any lymphocyte derived EV by eliminating events positive for CD3. EV negative for CD3 and CD41 (CD3/CD41-) were further analyzed for the presence of CD31, CD105, and CD144 to determine if they were endothelial derived EV (EEV). CD3/CD41- EV positive for CD31, CD105, or CD144 were referred to as EEV31, EEV105, and EEV144 respectively. To determine if EEV originated from CNS endothelial cells, MAL expression on EEV31, EEV105, and EEV144 was evaluated by pETX-647 binding. EEV31, EEV105, and EEV144 positive for pETX/MAL are referred to as CNS-EEV31, CNS-EEV105, and CNS-EEV144, respectively. A summary of these EV populations identified via flow cytometry and their marker expression is reviewed in Table 2. Enumeration of CNS-EEV31, CNS-EEV105, and CNS-EEV144 from our different patient groups revealed significant increases in the concentration of CNS-EEV in active RRMS patients compared to all other patient groups (Fig. 5C). When examining CNS-EEV31 concentrations, active RRMS patients had significantly higher concentrations than stable RRMS patients not receiving DMT, stable RRMS patients receiving natalizumab, and a trend towards significance compared to HC. When examining CNS-EEV105, active RRMS patients had significantly higher concentrations than all other patient groups. When examining CNS-EV144, active RRMS patients had significantly higher concentrations compared to HC and RRMS patients receiving natalizumab or ocrelizumab. Expression of CD31, CD105, and CD144 on EV was confirmed via western blot analysis of EV lysate from three separate donors (Fig. 5D). This indicates that active RRMS patients have increased levels of CNS-EEV31, CNS-EEV105, and CNS-EEV144 compared to HC and stable RRMS patients.

**Table 2:** Description of endothelial derived EV subpopulations identified in this study

EV Population	CD3/CD41	CD31	CD105	CD144	pETX/MAL +	Parental Cell
EEV31	−	+	−	−	−	endothelial
EEV105	−	−	+	−	−	endothelial
EEV144	−	−	−	+	−	endothelial
CNS-EEV31	−	+	−	−	+	CNS endothelial
CNS-EEV105	−	−	+	−	+	CNS endothelial
CNS-EEV144	−	−	−	+	+	CNS endothelial

+ or − indicates the presence or absence of cell-surface marker, respectively

We next wished to determine if individual CNS-EEV populations or individual EEV populations were more sensitive markers of active disease in RRMS patients. When examining the separate EEV populations, we observed an increase in EEV31, EEV105, and EEV144 concentrations in active RRMS patients compared to some of the other patient groups (Additional file 1: Fig. S2F–G). However, the statistical difference observed between levels of CNS-EEV31, CNS-EEV105, and CNS-EEV144 were more common and more significant than their EEV counterparts. In addition, we saw no significant difference in CD3/CD4 + EV concentrations (Additional file 1: Fig. S2E). Taken together, this data indicates that measurement of individual CNS-EEV populations is a more specific marker of disease activity in RRMS patients compared to total EV concentrations, individual EEV populations, or CD3/CD41+ EV populations.

**Fig. 6:**
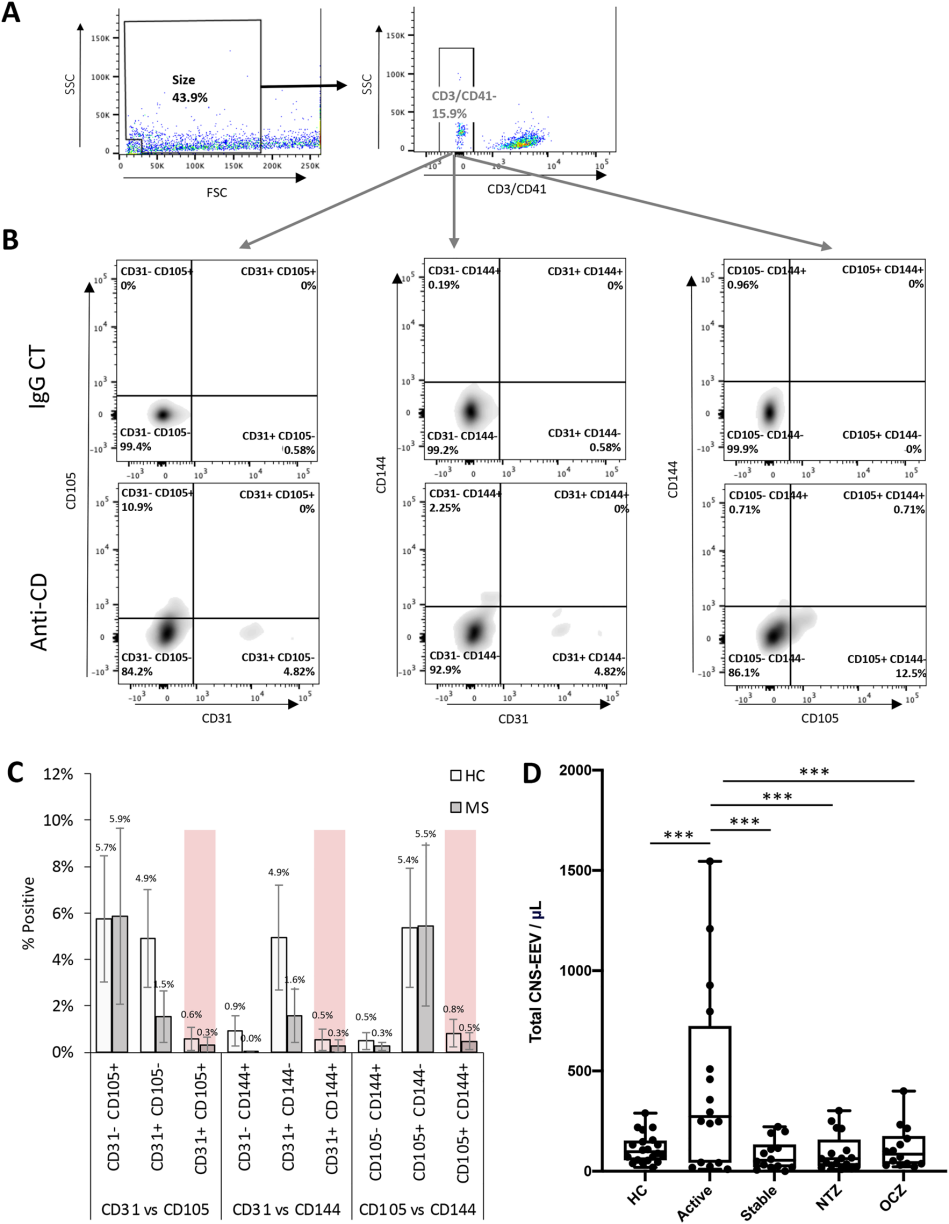
CNS-EEV populations are unique and are increased in RRMS patients with active disease. **A** Gating strategy for analysis of CD3/CD41- EV for expression of multiple endothelial markers. **B** Representative density plots of CD3/CD41- EV positive for CD31 versus CD105 (left), CD31 versus CD144 (center), or CD105 versus CD144 (right) when stained with anti-CD antibodies or appropriate isotype controls (IgG CT). **C** Percent of CD3/CD41- EV positive for indicated marker combinations. Results are expressed as a mean ± the STEDV of seven healthy controls (HC) or seven RRMS donors. Note that less than 1% of EV are positive for multiple markers, highlighted in red. **D** Total number of CNS-EEV per μL plasma was calculated by adding the levels of CSN-EEV31, CNS-EEV105, CNS-EEV144 for each separate donor. Calculated total CNS-EEV per μL of plasma for different patient groups including HC, active RRMS patients not receiving DMT (Active), stable RRMS patients not receiving DMT (Stable), or stable RRMS patients receiving natalizumab (NTZ) or ocrelizumab (OCZ). Results are displayed as box and whisker plots with each dot representing an individual donor. *** *p* ≤ 0.001 determined by one-way ANOVA with post-hoc Tukey HSD Test

We next wanted to determine if CNS-EEV31, CNS-EEV105, and CNS-EEV144 are unique EV populations or express multiple pan-endothelial markers. To achieve this, CD3/CD41- EV were analyzed for the presence of multiple endothelial markers by comparing expression of CD31 versus CD105, CD31 versus CD144, and CD105 versus CD144 (Fig. 6A, B). Examination of these events from seven HC and seven active RRMS patients revealed that the majority of EV only expressed a single endothelial marker (Fig. 4C). When comparing CD31 versus CD105 expression, only 0.3–0.6% percent of CD3/CD41- events were positive for both CD31 and CD105. When comparing CD31 versus CD144 expression, only 0.3–0.5% percent of CD3/CD41- events were positive for both CD31 and CD144. Finally, when comparing CD105 versus CD144, only 0.5–0.8% of CD3/CD41- events express both CD105 and CD144. This suggests that EEV31, EEV105, and EEV144 are unique populations and therefore CNS-EEV31, CNS-EEV105, and CNS-EEV144 are unique populations as well. We therefore reasoned that the total concentration of CNS-EEV in a single patient sample could be calculated by taking the sum of the individual CNS-EEV31, CNSEEV105, and CNS-EEV144 concentrations (Fig. 4D). This analysis revealed a significantly higher level of total CNS-EEV in active RRMS patients compared to all other patient groups. The same methodology was applied to determine the total EEV concentration (Additional file 1: Fig. S3). Active RRMS patients had significantly higher concentrations of total EEV compared to all other patient groups. However, the statistical difference observed between total CNS-EEV concentration was more significant than the differences seen between total EEV concentrations. For example, when examining total CNS-EEV concentrations, active RRMS were significantly higher versus HC (*p* = 0.001), stable RRMS receiving no DMT (*p* = 0.001), stable RRMS receiving natalizumab (*p* = 0.001), and stable RRMS patients receiving ocrelizumab (*p* = 0.001). In comparison, when examining total EEV concentrations, active RRMS were significantly higher versus HC (*p* = 0.02), stable RRMS receiving no DMT (*p* = 0.003), stable RRMS receiving natalizumab (*p* = 0.003), and stable RRMS patients receiving ocrelizumab (*p* = 0.001). Taken together, this data indicates that CNS-EEV concentrations are a more sensitive measurement of disease activity in MS patients compared to total EEV concentrations.

Finally, we wished to determine if total CNS-EEV concentrations were a more sensitive measurement of disease activity compared to the percentage of EV determined to be CNS-EEV (Additional file 1: Fig. S3). First, we examined the percent of total EV determined to be CNS-EEV (Additional file 1: Fig. S3A). To calculate this, we divided the total CNS-EEV concentration by the total EV concentration for each individual donor. No significant difference was observed between patient groups. Next, we examined the percent of total EEV that were determined to be CNS-EEV (Additional file 1: Fig. S3B). To calculate this, we divided the total CNS-EEV concentration by the total EEV concentration for each individual donor. No significant difference was observed between the different patient groups. Taken together, this data indicates that measurements of total CNS-EEV concentrations is a better measurement of disease activity in MS patients versus percentage calculations.

### Analysis of active RRMS patients

**Fig. 7:**
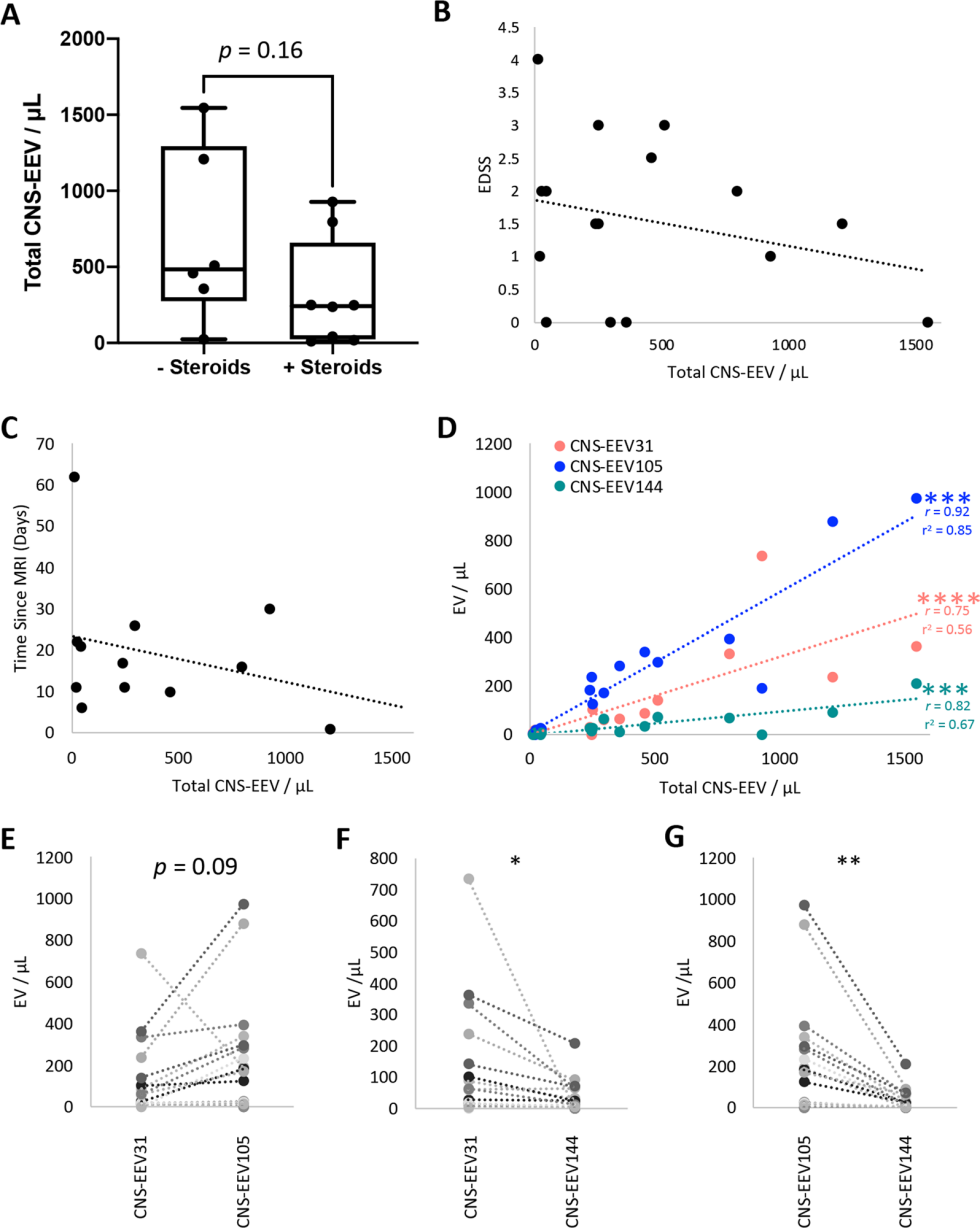
Detailed analysis of active RRMS patients. **A** Comparison of total CNS-EEV concentrations of active RRMS patients who had received steroids within 35 days prior to blood draw (+ steroids) (n = 6) or those who had not (- steroids) (n = 8). Results are displayed as box and whisker plots with each dot representing an individual donor. p value determined by unpaired students t-test. Correlation analysis of total CNS-EEV concentrations for active RRMS patients versus EDSS score (n = 16) (**B**) or days between MRI and blood draw (n = 11) (**C**)**. D** Correlation analysis of total CNS-EEV concentrations for active RRMS patients CNS-EEV31, CNS-EEV105, and CNS-EEV144 concentrations. *** *p* ≤ 0.001, *** *p* ≤ 0.0001 determined by Pearson’s r analysis. Pearson’s correlation coefficient (*r*) and r^2^ values are indicated in graphs for significant correlations. Paired t test plots for comparison of CNS-EEV31 versus CNS-EEV105 (**E**), CNS-EEV31 versus CNS-EEV144 (**F**), and CNS-EEV105 versus CNS-EEV144 (**G**) for individual active RRMS patients. Each pair represents an individual active RRMS patient. * *p* ≤ 0.05, ** *p* ≤ 0.01

Interestingly, when examining the total CNS-EEV concentrations in the active RRMS patient group, we observed a bimodal distribution (Fig. 6D) with 11 of the 16 (68.75%) patients with concentrations higher than 100 EV per μL of plasma and 5 out of the 16 patients (31.25%) with concentrations below 100 EV per μL of plasma. Although active RRMS patients were not receiving DMT at the time of blood draw, a portion of them had received steroids within 35 days of specimen collection (range one to 34 days). To determine if steroid use may influence the total CNS-EEV concentrations in these patients, we compared total CNS-EEV concentrations between active RRMS patients who had (+ steroids) or had not (- steroids) received steroid treatment within 35 days prior to specimen collection. Steroid use information was available for 14 out of the 16 active RRMS patients. Active RRMS patients who had received steroid treatment (n = 6) demonstrated a trend towards significantly lower total CNS-EEV concentrations than those who had not received steroid treatment (n = 8) (p = 0.16) (Fig. 7A). In addition, no correlation was observed between total CNS-EEV concentrations and individual patient EDSS (Fig. 7B) nor was a correlation observed between total CNS-EEV concentrations and the time elapsed between blood draw and MRI (Fig. 7C). MRI dates were available for 11 patients. Taken together this indicates that steroid use may influence total CNS-EEV concentrations, however our small sample size lacks sufficient power for a conclusive analysis and more testing is required.

Next, we wanted to determine if there was a consistent relationship between total CNS-EEV concentrations as well as individual CNS-EEV31, CNS-EEV105, and CNS-EEV144 concentrations in active RRMS patients. In other words, we wanted to know if CNS-EEV31 concentrations were low for an individual patient, would CNS-EEV105 and CNS-EEV105 concentrations also be low? To test for this, we performed a correlation analysis and paired t tests. We observed significantly strong, positive correlations between total CNS-EEV concentrations and CNS-EEV31 concentrations (*r* = 0.75, r^2^ = 0.56, and *p* = 0.0008), CNS-EEV105 concentrations (*r* = 0.92, r^2^ = 0.85, and *p* < 0.0001), ad CNS-EEV144 concentrations (*r* = 0.82, r^2^ = 0.67, and *p* = 0.0001) (Fig. 7D). Paired t test analysis also revealed a significant or close to significant relationship between CNS-EEV31 versus CNS-EEV105 concentrations (*p* = 0.09), CNS-EEV31 versus CNS-EEV144 concentrations (*p* < 0.05), and CNS-EEV105 versus CNS-EEV144 concentrations (*p* < 0.003) (Fig. 7E–G, respectively). Taken together, this indicates that CNS-EEV populations have a consistent relationship with one another in a single patient. In other words, if CNS-EEV31 concentrations are low for a single active RRMS patient, CNS-EEV105 and CNS-EV144 concentrations are typically low as well  and vice versa.

### Total CNS-EEV concentrations correlate with total EEV concentrations in RRMS patients

**Fig. 8:**
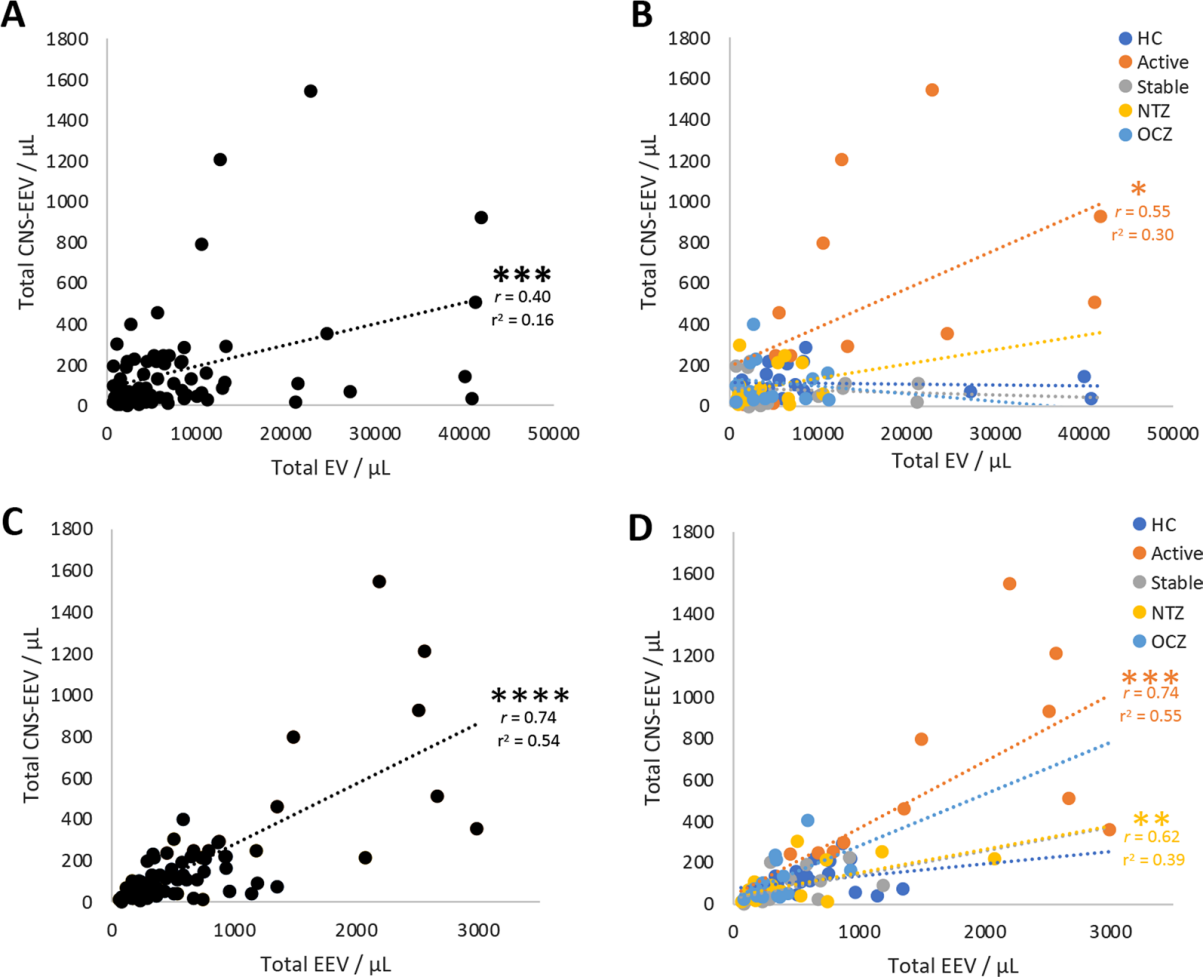
CNS-EEV correlation analysis. Correlation analysis of total EV concentrations versus total CNS-EEV concentrations of all donors (**A**) or when separated into different patient groups including (**B**) HC, active RRMS patients not receiving DMT (Active), stable RRMS patients not receiving DMT (Stable), or stable RRMS patients receiving natalizumab (NTZ) or ocrelizumab (OCZ). Note the data in C and D are identical but have been analyzed differently. Correlation analysis of total EEV levels versus total CNS-EEV levels for all donors (**C**) or when broken into different patient groups (**D**) as previously described. Note the data in C and D are identical but have been analyzed differently. * *p* ≤ 0.05, ** *p* ≤ 0.01, *** *p* ≤ 0.001, **** *p* ≤ 0.0001 determined by Pearson’s r analysis. Pearson’s correlation coefficient (*r*) and r^2^ values are indicated in graphs for significant correlations

To determine if the total CNS-EEV concentrations correlated with the total EV concentrations in all patient groups, we performed a correlation analysis examining all donors as a single group (Fig. 8A). When examining all donors as a single group, total CNS-EEV concentrations demonstrated a moderate, but significant positive correlation with total EV concentrations (*r* = 0.40, r^2^ = 0.16, and *p* = 0.0002)(Fig, 8A). However, when donors were separated into their individual patient groups, only active RRMS patients demonstrated a positive correlation between total CNS-EEV and total EV concentrations (*r* = 0.55, r^2^ = 0.30, and *p* = 0.03) (Fig. 8B). This indicates that an increase in total CNS-EEV concentrations correlates with an increase in total EV concentrations in active RRMS but not HC or stable RRMS patients.

To determine if the total CNS-EEV concentrations correlated with total EEV concentrations, we performed a correlation analysis examining all donors as a single group (Fig. 8C). When examining all donors as a single group, total CNS-EEV concentrations demonstrated a strong and significant positive correlation with total EEV concentrations (*r* = 0.74, r^2^ = 0.54, and *p* < 0.0001) (Fig. 8C). When donors were separated into their individual patient groups, a similar trend was observed for all RRMS groups but not HC (Fig. 8B). Active RRMS demonstrated a significantly strong correlation (*r* = 0.74, r^2^ = 0.55, and *p* = 0.001) while stable RRMS patients on natalizumab had a moderate but significant correlation (*r* = 0.62, r^2^ = 0.39, and *p* = 0.007). Stable RRMS patients not receiving DMT (*r* = 0.46, r^2^ = 0.21, and *p* = 0.09) and stable RRMS patients receiving ocrelizumab (*r* = 0.50, r^2^ = 0.25, *p* = 0.07) both demonstrated a moderate correlation approaching significance. Taken together, this data indicates that CNS-EEV concentrations positively correlate with total EEV concentrations in RRMS patients but not HC.

### Total CNS-EEV concentrations do not depend on patient age but may be influenced by gender

**Fig. 9:**
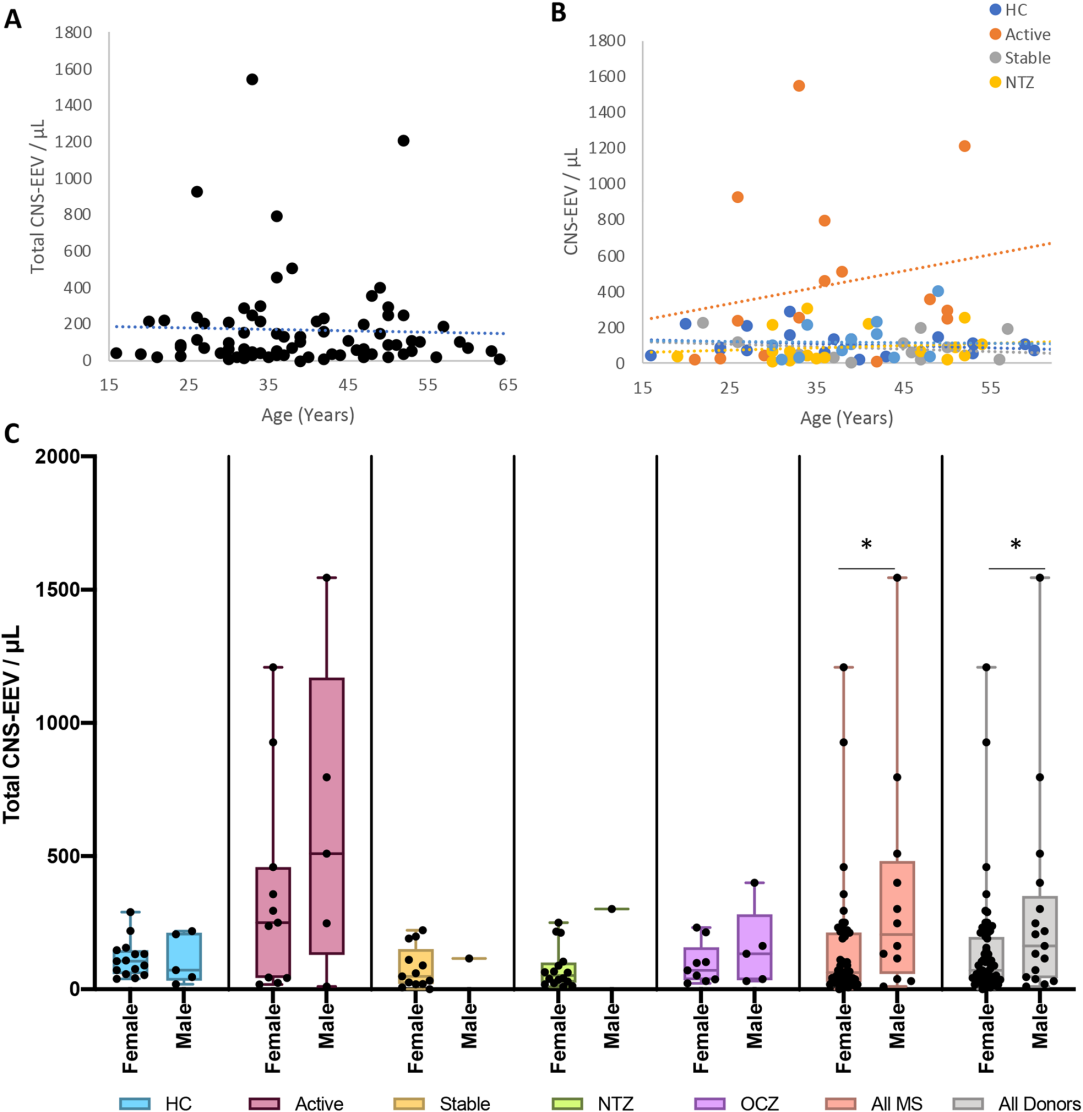
Age and gender analysis for total CNS-EEV concentrations. Correlation analysis of patient age in years and total CNS-EEV concentration for all donors as a single group (**A**) or when separated into different patient groups (**B**). Note the data in A and B are identical but have been analyzed differently. Patient groups include HC, active RRMS patients not receiving DMT (Active), stable RRMS patients not receiving DMT (Stable), or stable RRMS patients receiving natalizumab (NTZ) or ocrelizumab (OCZ). No significant correlations were determined using Pearson’s r analysis. (**C**) Comparison of total CNS-EEV concentrations in female or male donors for indicated patient groups. Analysis was also performed when all MS patients were grouped together (All MS) or when all donors including HC were grouped together (All Donors). Results are displayed as box and whisker plots with each individual patient represented as a dot. * *p* ≤ 0.05 determined by unpaired student’s t-tests

To determine if patient age influences total CNS-EEV concentrations, a correlation analysis was performed examining at all donors as a single group or when donors were separated into their individual patient groups (Fig. 9A and B). No significant correlation was observed. This indicates that age does not influence total CNS-EEV concentrations.

To determine if gender influences total CNS-EEV concentrations, total CNS-EEV concentrations were compared between female and male donors in the individual patient groups (Fig. 9C). This analysis revealed no significant difference between males and females. However, this may be due to the low number of men enrolled in this study. To try and overcome this, we compared total CNS-EEV concentrations when all RRMS patients were grouped together or when all donors including HC were grouped together. This analysis revealed that male donors had significantly higher levels of total CNS-EEV compared to female donors when all RRMS donors were grouped together and when all donors including HC were grouped together. This indicates that men with RRMS may have higher levels of total CNS-EEV compared to females with RRMS. However, these results are only preliminary due to small sample size.

## Discussion

To the best of our knowledge, this is the first time CNS-EEV or EV derived from the BBB have been identified in circulation from humans. To achieve this, we developed a novel method to identify EV originating from CNS endothelial cells by detection of multiple, cell-specific markers on EV isolated from patient plasma via flow cytometry. First, lymphocyte or platelet derived EV are excluded from analysis via detection of CD3 or CD41, respectively. EEV are then detected via expression of pan-endothelial markers CD31, CD105, or CD144. Finally, EEV positive for CD31, CD105, or CD144 are determined to be of CNS-endothelial origin via expression of MAL, a cell surface protein recently identified to be specifically expressed on CNS endothelial cells [62, 63]. Using this method, we identified three distinct CNS-EEV populations including CNS-EEV31, CNS-EEV105, and CNS-EEV144. These EV populations and their individual protein expression is summarized in Table 2. In our pilot study examining the circulating levels of CNS-EEV from 81 blood donors, we determined that CNS-EEV concentrations are higher in RRMS patients with active disease compared to HC, stable RRMS patients not receiving DMT, stable RRMS patients not receiving natalizumab, and stable RRMS patients receiving ocrelizumab. This includes an increase in total CNS-EEV as well as individual CNS-EEV31, CNS-EEV105, and CNS-EEV144 populations. Interestingly, natalizumab and ocrelizumab treatment did not appear to significantly alter CNS-EEV levels between stable RRMS patients. In comparison, active RRMS patients not receiving DMT but who had received steroid treatment within 35 days of blood draw may have lowered total CNS-EEV concentrations compared to active patients who had not, indicating that CNS-EEV levels may be useful biomarkers of treatment efficacy. However, we recognize this conclusion is limited based on our small sample size and requires more investigation. Taken together, this data indicates that plasma concentrations of CNS-EEV may be biomarkers of active disease in MS patients. Because CNS-EEV are derived from the endothelial cells of the CNS, we hypothesize that increased CNS-EEV concentrations are indicators of CNS endothelial dysfunction including BBB permeability. This is supported by (1) a handful of previous publication that have reported an increase in BBB derived EV by detection of tight junction proteins occludin and claudin-5 in mouse models with known BBB permeability [99–102] and (2) the observation that 14 out of our 16 active RRMS patients have confirmed BBB permeability via MRI detection of CEL.

Although previous publications have indicated an increased level of circulating EEV in MS patients compared to HC [44, 50, 51, 53, 55], our data indicates that examination of CNS-EEV rather than EEV populations may be a more sensitive method for detecting disease activity in MS patients. In previous studies, EEV were identified by expression of a single pan-endothelial marker including CD31 [50], CD146 (MCAM) [44], CD62E (E-Selectin) [51, 55], or CD105 [53]; or expression of CD31 in the absence of CD42 [51, 54]. Using our techniques, we confirmed an increase in total EEV concentrations between active RRMS patients versus all other patient groups. However, significant differences were more common and more significant when comparing CNS-EEV populations versus EEV populations. Although previous authors have surmised that increased EEV levels in MS patients may be indicative of BBB permeability, increased levels of EEV are not specific to MS and may confound results. Other common comorbidities like diabetes and heart disease have known endothelial pathologies and increased levels of circulating EEV [81–83, 103, 104]. Therefore, increased levels of EEV are not a specific marker of BBB dysfunction and are not necessarily disease specific. By concentrating on CNS-EEV levels however, we can focus more specifically on CNS endothelial injury/activation including BBB permeability, a critical component of MS lesion pathophysiology [28–30, 59, 60].

To identify EV originating from CNS endothelial cells, we relied on detection of the myelin and lymphocyte protein MAL on EV expressing pan-endothelial markers CD31, CD105, and CD144. Detection of MAL expression was chosen because it appears that MAL is specifically expressed by CNS endothelial cells compared to endothelial cells of peripheral organs, at least in mice [62, 63]. Importantly, MAL expression in human brain endothelial cells has been confirmed in transcriptional studies [64–66]. Although human MAL has been demonstrated to bind pETX [98], the selective expression of the MAL protein in human brain endothelial cells compared to peripheral endothelial cells needs still needs to be evaluated. MAL is a highly hydrophobic proteolipid, and its expression is limited to T-lymphocytes, myelin producing oligodendrocytes, polarized epithelial cells, and endothelial cells of the CNS [62, 97, 105–114]. Although MAL expression in endothelial cells appears to be limited to the CNS, its function in these cells is unknown. In other MAL expressing cells, MAL is known to play an important role in apical protein sorting and lipid raft formation and maintenance [114–123]. It seems reasonable that MAL would play a similar role in endothelial cells and may even be a critical component in maintaining the polarized phenotype observed in endothelial cells of the BBB [124, 125].

To detect EV MAL expression, we utilized binding of the MAL-specific ligand, pETX [98, 126], as there is no currently available anti-MAL antibody suitable for flow cytometry. This ligand was selected based on numerous, independent studies that strongly indicate MAL is the receptor for pETX [62, 97, 98, 126–132]. MAL has been repeatedly demonstrated by independent groups to be both necessary and sufficient for pETX binding [62, 97, 98, 126–129]. Several publications have demonstrated that knock out of MAL expression in pETX sensitive organisms or cell lines totally abolishes pETX binding and/or toxicity while exogenous expression of MAL confers pETX binding in normally resistant cells or organisms [62, 97, 98, 126–129]. In addition, the specificity of pETX for MAL is supported by descriptive studies that strongly correlate pETX binding to MAL expression in multiple sensitive cell lines [130–132]. Finally, direct protein–protein interaction between pETX and MAL have been published by two independent groups via immunoprecipitation and affinity purification column [126, 129]. Taken together, this strongly indicates that pETX is a specific ligand for MAL expression. However, we recognize that pETX may have other, yet identified receptors, and therefore pETX binding to EV may not be exclusively indicative of MAL expression.

While CD31, CD105, and CD144 have been demonstrated to be expressed on EEV [61], expression of MAL on circulating EV has not been evaluated. Interestingly, MAL may play an important role in EV biogenesis, as knockout of MAL in T-cells significantly impairs exosome secretion [120]. Considering the importance lipid rafts play in EV biogenesis [133, 134], it seems probable that MAL’s role in EV biogenesis may be related to its role in lipid raft organization and apical protein sorting.

Interestingly, CNS-EEV populations appear to be unique, as the majority of CNS-EEV appear to express a single pan-endothelial marker (CD31 CD105, or CD144). The reason for this is unclear. It may be possible that EV only express a limited amount of surface proteins. Alternatively, detection of multiple markers on a single EV may be due to steric hindrance or limited signal when using antibodies to detect proteins on particles with a small surface area. However, it is tempting to speculate that increases in specific CNS-EEV populations may be indicative of specific, pathogenic mechanisms occurring at the BBB. For example, in vitro experiments have demonstrated that stimulation of endothelial cells with different agents results in differential protein and miRNA packaging into secreted EEV and are representative of the activation state of the parental cell [55, 135–137]. Therefore, an increase in a specific CNS-EEV populations with a specific protein content may reflect specific changes occurring at the BBB. For example, histopathological examination of MS lesions has revealed an increase in vasculature CD31 and CD105 expression. Hence, an increase in circulating CNS-EEV31 or CNS-EEV105 concentrations may be reflective of the cellular changes occurring in the endothelial of MS lesions. Because EV reflect the biological state of their parental cells [55, 135–141], molecular analysis including miRNA profiling of purified CNS-EEV may also provide endothelial-specific mechanisms of pathogenesis, as bulk EV analysis have indicated significant differences in miRNA expression in MS derived EV versus HC [142–147]. These studies are ongoing.

Our SEC isolation of EV from patient plasma method was optimized for detection of EV via flow cytometry. Based on analysis of submicron polystyrene beads, we estimate we are detecting EV ranging in size from 0.5 to 1 μm, with some limited detection of 0.2 μm EV. This is consistent with previously published results using conventional flow cytometry to analyze EV [85–87]. However, TEM and NTA analyses reveal a significant amount of EV smaller than 0.2 μm contained within our SEV isolated samples. Therefore, our flow cytometry analysis is only detecting a small portion of our total EV population. However, flow cytometry is the most acceptable method for analysis of multiple surface markers on a single EV. Current studies are underway to improve detection of multiple markers on all EV within our SEC fractions of interest.

## Conclusion

In summary, this work builds on the small but growing amount of evidence that CNS derived EV can be detected in peripheral circulation of MS patients and may be useful biomarkers of specific disease activity [46, 47]. Taken together, this indicates that analysis of circulating CNS derived EV including CNS-EEV may be helpful biomarkers of specific disease activity in MS patients.

## Supplementary Information

**Additional file 1: Figure S1**. Gating strategy and enumeration of individual EEV populations. **Figure S2**. Enumeration of total EEV. **Figure S3**. Percentages of total CSN-EEV do not differ between patient groups. **Table S1**. Flow cytometry antibodies. **Table S2**. Western blot antibodies, and Supplemental Materials and Methods consisting of detailed experimentation.

## Data Availability

All data and material are available upon from the corresponding author upon request.

## Abbreviations

BBB: Blood–brain barrier
CEL: Contrast enhancing lesions
CSF: Cerebral spinal fluid
CNS-EEV: CNS endothelial derived extracellular vesicles
CNS-EEV31: CNS endothelial derived extracellular vesicles expressing CD31 and MAL
CNS-EEV105: CNS endothelial derived extracellular vesicles expressing CD105 and MAL
CNS-EEV144: CNS endothelial derived extracellular vesicles expressing CD144 and MAL
EV: Extracellular vesicles
EEV: Endothelial derived extracellular vesicles
EDSS: Expanded Disability Status Scale
MAL: Myelin and lymphocyte protein
FSC: Forward scatter
DMT: Disease modifying therapy
HC: Healthy controls
HUVEC: Human umbilical cord vein endothelial cell
NTA: Nanoparticle tracking analysis
PBS: Phosphate buffered saline
PFP: Platelet free plasma
PPMS: Primary progressive multiple sclerosis
RRMS: Relapsing remitting multiple sclerosis
RT: Room temperature
SPMS: Secondary progressive multiple sclerosis
SEC: Size exclusion column/chromatography
SSC: Side scatter
TBS-T: Tris Buffered Saline with Tween 20
TEM: Transmission electron microscopy
