# Supplementary material for: CNS endothelial derived extracellular vesicles are biomarkers of active disease in multiple sclerosis
Source: Fluids Barriers CNS. 2022 Feb 8;19:13. doi: 10.1186/s12987-021-00299-4 (PMC8822708; doi:10.1186/s12987-021-00299-4)
Supplement: Supplementary file 1 — Additional file 1: Figure S1. Gating strategy and enumeration of individual EEV populations. Figure S2. Enumeration of total EEV. Figure S3. Percentages of total CSN-EEV do not differ between patient groups. Table S1. Flow cytometry antibodies. Table S2. Western blot antibodies, and Supplemental Materials and Methods consisting of detailed experimentation. [file 12987_2021_299_MOESM1_ESM.docx]

# Supplemental Figures

**Supplemental Figure 1 Gating strategy and enumeration of individual EEV populations.** Representative dot plots when SEC isolated EV stained with fluorescently labeled anti-CD3, anti-CD antibodies (right) or appropriate isotype controls (IgG CT, left). Positive gates were selected based on fluorescence of IgG CT-stained controls. **(A)** Representative dot blots for fluorescent markers anti-CD3 and anti-CD41 (CD3/CD41). Events negative for CD3/CD41 (CD3/CD41-) were analyzed for expression of **(B)** CD31, **(C)** CD105, or **(D)** CD144 when stained with anti-CD markers (right) or isotype controls (IgG CT, left). Positive gates were adjusted for each individual donor. Enumeration of EEV-31 **(E)**, EEV105 **(F)**, and EEV144 **(G)** per ul of plasma different patient groups HC, active RRMS patients not receiving DMT (Active), stable RRMS patients not receiving DMT (Stable), or stable RRMS patients receiving natalizumab (NTZ) or ocrelizumab (OCZ). Results are displayed as box and whisker plots with each individual patient represented as a circle. * *p* ≤ 0.05, ** *p* ≤ 0.01, *** *p* ≤ 0.001 determined by one-way ANOVA with post-hoc Tukey HSD Test.

**Supplemental Figure 2 Enumeration of total EEV.** As depicted in Figure 6, less than 1% of EV are positive for multiple markers pan-endothelial markers CD31, CD105, and CD144. This indicates that EEV only express a single endothelial maker, indicating EEV31, EEV105, and EEV144 are unique populations. Therefore, the total number of EEV per ul plasma was calculated by adding the levels of EEV31, EEV105, EEV144 for each separate donor. Calculated total EEV per ul of plasma for different patient groups including HC, active RRMS patients not receiving DMT (Active), stable RRMS patients not receiving DMT (Stable), or stable RRMS patients receiving natalizumab (NTZ) or ocrelizumab (OCZ). Results are displayed as box and whisker plots with each dot representing an individual donor. * *p* ≤ 0.05, ** *p* ≤ 0.01 determined by one-way ANOVA with post-hoc Tukey HSD Test.

**Supplemental Figure 3 Percentages of Total CSN-EEV do not differ between patient groups. (A)** To calculate the percent of Total EV, the total CNS-EEV concentration was divided by the total EV concentration for each individual donor. **(B)** To calculate the percent of Total EEV, the total CNS-EEV concentration was divided by the EEV concentration for each patient. Patient groups include HC, active RRMS patients not receiving DMT (Active), stable RRMS patients not receiving DMT (Stable), or stable RRMS patients receiving natalizumab (NTZ) or ocrelizumab (OCZ). Results are displayed as box and whisker plots with each individual patient represented as a dot. No significant differences between groups were observed as determined by one-way ANOVA with post-hoc Tukey HSD Test.

**Supplemental Tables**

**Supplemental Table 1 Flow cytometry antibodies**

| **Group** | **Target** | **Conjugate** | **Manuf.** | **PN** | **Dilution** | **Isotype CT** | **Manuf.** | **PN** |
| --- | --- | --- | --- | --- | --- | --- | --- | --- |
| Common EV | CD9 | PE | BioLegend | 312105 | 1:50 | Mouse IgG1, κ | BioLegend | 400112 |
|  | CD63 | FITC | BioLegend | 353005 | 1:100 | Mouse IgG1, κ | BioLegend | 400109 |
|  | CD81 | Pacific Blue | BioLegend | 349515 | 1:50 | Mouse IgG1, κ | BioLegend | 400131 |
| CNS-EEV | CD3 | Pacific Blue | BioLegend | 300330 | 1:100 | Mouse IgG2a, κ | BioLegend | 400235 |
|  | CD31 | APC/Cy7 | BioLegend | 303120 | 1:50 | Mouse IgG1, κ | BioLegend | 400127 |
|  | CD41 | Pacific Blue | BioLegend | 303714 | 1:100 | Mouse IgG1, κ | BioLegend | 400131 |
|  | CD105 | AF-488 | BioLegend | 323210 | 1:50 | Mouse IgG1, κ | BioLegend | 400132 |
|  | CD144 | PE | BioLegend | 348506 | 1:50 | Mouse IgG2a, κ | BioLegend | 400211 |

**Supplemental Table 2 Western blot antibodies**

| **Target** | **Manuf.** | **PN** | **Host** | **Dilution** |
| --- | --- | --- | --- | --- |
| GM130 | Cell Signaling Technology | 12480 | rabbit | 1:1000 |
| Flotillin-1 | Cell Signaling Technology | 18634 | rabbit | 1:1000 |
| CD9 | Cell Signaling Technology | 13174 | rabbit | 1:1000 |
| CD63 | Abcam | ab134045 | rabbit | 1:5000 |
| CD31 | Abcam | ab76533 | rabbit | 1:10,000 |
| CD105 | Abcam | ab169545 | rabbit | 1:2000 |
| CD144 | Abcam | ab33168 | rabbit | 1:5000 |

**Supplemental Materials and Methods**

**Blood collection and plasma separation:** Peripheral blood was harvested via venipunture of the median cubital vein into 4.0ml BD vacutainers containing K2 EDTA 7.2mg (PN: 367861). Typically, a total of 16-32ml of blood (four to eight tubes) were harvested per patient. The time of day when blood was harvested, pre/postprandial status, and recent exercise levels were not recorded. Blood was stored at room temperature (RT) until processing and was processed within six hours of collection. Note, it is critical that blood be stored at RT instead of 4°C to prevent platelet activation and subsequent release of platelet EV. To separate plasma from cellular components, vacutainers were spun at 450 *g* in an Eppendorf Centrifuge 5810 R equipped with an A-5-62 rotor and swinging buckets for ten minutes at RT. Again, it is of vital importance that blood and plasma be centrifuged at RT instead of 4°C to prevent platelet activation and subsequent release of platelet EV. Plasma was harvested and transferred to 15ml conical tubes. Harvested plasma was spun at 2000g for ten minutes at RT and then transferred to a fresh 15ml conical tube, discarding the pellet. The plasma was spun again at 2000g for ten minutes at RT to remove any additional platelets. After the two 2000g spins, the platelet free plasma (PFP) was transferred to a single 15ml conical tube and mixed gently by inverting. The PFP was then separated into 1ml aliquots and stored in 2.0ml cryogenic vials (Corning, PN:431416) at -80°C. 1ml aliquots of PFP were thawed for two minutes in a 37°C water bath prior to isolation via SEC.

**Preparation of phosphate buffered saline and flow cytometry sheath fluid:** Phosphate buffered saline (PBS) (VWR, PN: 97062-950) was prepared with deionized, ultrapure water prepared using a Milli-Q Direct-Q 3 Water Purification System. All PBS and flow cytometry sheath fluid (BD FACSFlow, PN: 342003) was filtered through a vacuum filtration unit with a 0.2um a polyethersulfone membrane (VWR, 10040-440) prior to use. Only filtered PBS and sheath fluid were used for experiments.

**Isolation of size exclusion columns (SEC) fractions for flow cytometry analysis:** EV were isolated from 1ml of thawed PFP using Izon qEV 70nm size exclusion columns (SEC) (Fisher Scientific, PN: NC1507069) equilibrated to RT. To prepare the SEC, the shipment liquid was allowed to drain from the SEC via gravity and discarded. The SEC was then washed with 10mls of PBS via gravity and the flow-through discarded. 1ml of the thawed PFP was then applied directly to the column and allowed to fully absorb. The subsequent 1ml flow-through was discarded. Next, a total of 6mls of PBS was added to the column in 0.5ml, sequential aliquots. The flow-through was collected in 0.5ml sequential fractions in microcentrifuge tubes and then analyzed via flow cytometry. SEC columns were not reused.

**Isolation of EV from PFP via SEC for patient analysis:** EV were isolated from 1ml of thawed PFP using Izon qEV 70nm size exclusion columns (SEC) (Fisher Scientific, PN: NC1507069) equilibrated to RT. To prepare the SEC, the shipment liquid was allowed to drain from the SEC via gravity and discarded. The SEC was then washed with 10mls of PBS via gravity and the flow-through discarded. 1ml of the thawed PFP was then applied directly to the column and allowed to fully absorb. The subsequent 1ml flow-through was discarded. 3mls of PBS was then added and the 3ml of flow-through discarded. Finally, 1.5ml of PBS was added to the column, and the 1.5ml of flow through containing our EV of interests harvested in a microcentrifuge tube. Isolated EV were used immediately for analysis. Any remaining EV solution was stored at -20°C. SEC columns were not reused.

**Nanoparticle Tracking Analysis (NTA)**: SEC isolated EVs size distributions were characterized by NTA using a Malvren NanoSight NS500 instrument equipped with an Andor EM-CCD camera. As a quality control, analysis of polystyrene beads of known size (National Institute of Standards and Technology) were immediately analyzed prior to EV analysis. For optimal analysis, SEC isolated EVs were pre-diluted to achieve a concentration within the 10^8^ to 10^9^ vesicles per ml in PBS. Video acquisitions were performed with NTA software v3.1 using a camera level below 12 and gain level of 1 for EVs and polystyrene beads. Three replicate videos of 60 seconds were captured per sample in separate locations of sample solution.

**High Speed Centrifugation Experiments**: EV were isolated from 1ml PFP via SEC as previously described in a total of 1.5ml PBS. To pellet EV ~150nm and larger, 250ul of isolated EV solution was centrifuged at 18,000g at 4°C for one hour in 1ml, open-top, thick wall polypropylene tube (Beckman Coulter, PN: 347287) using a benchtop centrifuge (Beckman Coulter, Microfuge 18, PN: 367160). To pellet EV ~30nM and larger, 250ul of isolated EVs in PBS were centrifuged at 100,000gfor one hour at 4C in 1°ml, open-top, thick wall polypropylene tube (Beckman Coulter, PN: 347287) using a tabletop ultracentrifuge (Beckman Coulter, Optima™ Max-XP Tabletop Ultracentrifuge, PN: 393315) using a fixed angle rotor (Beckman Coulter, MLA-130 Rotor, PN: 367114). Supernatants were carefully removed and saved for further analysis and the pellets resuspended in 250ul PBS.

**Flow cytometry settings for analysis of submicron particles:** All samples were analyzed using a BD FACSVerse flow cytometer equipped with a BD Flow sensor. The Flow sensor is an inline sensor that directly measures the flow rate of particles and therefor sampled volume, providing accurate absolute counts. A three-laser configuration was used (488 nm, 640 nm, and 405 nm) with the manufacturer settings. BD FACSuite CS&T research beads (PN: 650621) were used daily to calibrate and perform quality control checks per the manufacturer’s instructions. All samples were analyzed in 5ml polystyrene round bottom tubes (Falcon, PN: 352054) using the manual port or BD FACS Universal Loader equipped with a 40-tube rack. All samples were acquired using medium sample flow rates (60ul/min) with a normal sheath core stream fluid velocity (5.5 m/s). Data was collected using BD FACSuite software and exported to BD Flowjo software for analysis. Event counts and volume metrics were exported to Microsoft Excel for final calculations. Invitrogen’s Flow Cytometry Size Kit containing nonfluorescent polystyrene microspheres ranging in size from 1-15um Calibration Kit (Thermo Fischer Scientific, PN: F13838), and Invitrogen’s Flow Cytometry Submicron Particle Size Reference Kit containing green-fluorescent polystyrene microspheres ranging in size from 0.02-2um (Thermo Fischer Scientific, PN: F13839,) were used to determine appropriate settings for submicron analysis. Beads were diluted in PBS prior to analysis. FSC and SSC voltage was adjusted to 765 and 465, respectively, and resulted in reliable detection of 1um particles. An FSC threshold of 10,000 was used.

**Staining protocol for flow cytometry analysis**: EV were isolated from 1ml PFP via SEC as previously described in a total of 1.5ml PBS. The EV concentration was determined by analysis using a BD FACSVerse flow cytometer equipped with a flow sensor using the settings described previously. Data was collected using BD FACSuite software and exported to BD Flowjo software for analysis. Event counts and volume metrics were exported to Microsoft Excel for final calculations. The size gate was determined by running Thermo Fischer Scientific 1um beads (Fig 1). All event counts were based on the number of events within the size gate. SEC isolated EV were adjusted to 500 events/ul in PBS to ensure consistent staining conditions and the dilution factor recorded. 50ul of diluted EV solution were transferred to 5ml polystyrene round bottom tubes (Falcon, PN: 352054) for staining. A list of antibodies used for staining, staining dilutions, and appropriate isotype controls are listed in Supplemental Table 1. Staining dilutions were determined via titration and comparison of signal to noise ratio versus isotype controls. Prior to staining, all antibodies and pETX-647 stock solutions were centrifuged at 18,000g on a tabletop centrifuge for ten minutes to pellet any aggregates. As a negative control, pETX-647 was pretreated with an anti-ETX neutralizing antibody, JL004, at 1mg/ml for 20 minutes at 37°C to prevent binding. For analysis of common EV markers, diluted EV were stained using the following conditions 1) unstained control, 2) isotype stained control (quadruplicate), and 3) anti-CD markers (quadruplicate). For analysis of CNS-EEV markers, diluted EV were stained using the following conditions: 1) unstained control, 2) isotype-stained control, 3) anti-CD markers plus pETX-647 to detect MAL expression (in triplicate), and 4) anti-CD markers plus pETX-647 pretreated with anti-ETX antibody JL004 (in triplicate). EV were stained for two hours at RT with gentle agitation. After incubation, stained EV were diluted with 500ul of PBS (1:11 dilution), mixed well, and analyzed via flow cytometry. The final concentration for flow cytometry analysis is 50 events/ul, which was determined to be an optimal concentration to prevent swarming artifacts during analysis. Data was collected using BD FACSuite software and exported to BD Flowjo software for analysis. To determine gates positive for CD3, CD31, CD41, CD105, and CD144 expression, EV stained with isotype controls were used as negative controls for each individual donor (Supplemental Fig 1A-D). To determine gates positive for MAL expression, EV stained with pETX-647 pretreated with JL004 were used as negative controls for each individual donor (Fig. 4A and B). Event counts and volume metrics were then exported to Microsoft Excel for final calculations. Because each donor is stained in three to six replicates, the individual EV population counts are determined from the mean of these replicates for each individual donor. Plasma concentrations were determined using the calculated means, collected volume metrics adjusted to 50 events/ul, and dilutions used during staining and SEC isolation. All results are reported as events per ul of plasma unless otherwise noted.

**Transmission Electron Microscopy (TEM) Analysis**: Three to five ul of SEC isolated EV were allowed to settle for one minute onto Glow-discharge Formvar and carbon-coated 400 mesh copper grids (Electron Microscopy Sciences) Excess sample was wicked from grid using a #1 Whatman filter paper. Immediately after, four consecutive drops of 1.5% aqueous uranyl acetate were added to the grid, wicking excess moisture between each drop as described. Grids were allowed to air dry five minutes before imaging. Samples were viewed on a JEM-1400 transmission electron microscope (JEOL, USA, Inc., Peabody, MA) operated at 100 kV and images were captured on a Veleta 2K x 2K CCD camera (EM-SIS, Germany).

**Western blot analysis:** EV were isolated from 1ml PFP via SEC as previously described in a total of 1.5ml PBS. Approximately 1ml of isolated EV was centrifuged at 100,000gfor one hour at 4°C in 1ml, open-top, thick wall polypropylene tube (Beckman Coulter, PN: 347287) using a tabletop ultracentrifuge (Beckman Coulter, Optima™ Max-XP Tabletop Ultracentrifuge, PN: 393315) using a fixed angle rotor (Beckman Coulter, MLA-130 Rotor, PN: 367114). Supernatants was carefully removed and the pellet was resuspend in 100-300ul radioimmunoprecipitation assay (RIPA) buffer (Thermo Scientific, PN: 89901) containing Halt Protease and Phosphatase Inhibitor (Thermo Scientific, PN: 78440) for ten minutes on ice and stored a -20°C until use. HUVEC lysate (Novus Biologicals, PN: NBP2-13174) were used as controls. Protein concentration was determined by Pierce BCA Protein Assay Kit (Thermo Scientific, PN: 23227). 2ug of lysates were diluted into 4x Laemmli Sample Buffer (Bio-Rad, PN: 1610747) containing 5% 2-Mercaptoethanol (Bio-Rad, PN: 1610710) and heated at 95°C for five minutes. 2ug of prepared lysates were loaded onto 4–20% Mini-PROTEAN TGX Stain-Free gels (Bio-Rad, PN:4561094). Gels were run in Tris/Glycine SDS running buffer (Bio-Rad, PN:1610732) at 200 volts for 35 min. Semi-dry transfers were performed in Tris/Glycine transfer buffer (Bio-Rad, PN:1610734), using the Bio-Rad Trans-Blot SD Semi-Dry Electrophoretic Transfer Cell system at 15 volts for 15 min. Blots were blocked in 5% Blotting-Grade Blocker nonfat milk (Bio-Rad, PN: 1706404) in Tris Buffered Saline with Tween 20 (TBS-T)(Bio-rad, PN: BUF028) for one hour at room temperature. Blots were then incubated with primary antibodies (Supplemental Table 2) in blocking solution overnight at 4°C. Blots were washed three times for five minutes in TBS-T at room temperature and incubated with secondary antibody peroxidase-conjugated Affinipure Goat Anti-Rabbit IgG H + L (Jackson ImmunoResearch, 711-035-152) at 0.024 µg/mL (1:50,000) in blocking solution for two hours at room temperature. Blots were washed three times for five min in TBS-T and developed for five min at room temperature in Clarity Max Western ECL Substrate (Bio-rad, PN:1705062). The developed blots were visualized on 5 × 7 CL-XPosure Films (Thermo Fisher Scientific, PN: 34090) at various exposure times using a Konica Minolta SRX-101A film processor.
